# Hydrothermally Grown Dual‐Phase Heterogeneous Electrocatalysts for Highly Efficient Rechargeable Metal‐Air Batteries with Long‐Term Stability

**DOI:** 10.1002/advs.202203663

**Published:** 2022-09-14

**Authors:** Chandran Balamurugan, Changhoon Lee, Kyusang Cho, Jehan Kim, Byoungwook Park, Yusin Pak, Jaemin Kong, Sooncheol Kwon

**Affiliations:** ^1^ Department of Energy and Materials Engineering Dongguk University‐Seoul Seoul 04620 Republic of Korea; ^2^ Heeger Center Advanced Materials (HCAM) Gwangju Institute of Science and Technology (GIST) Gwangju 500‐712 Republic of Korea; ^3^ Max Planck POSTECH Center for Complex Phase of Materials Pohang University of Science and Technology Pohang 37673 Korea; ^4^ Research Institute for Solar and Sustainable Energies (RISE) Gwangju Institute of Science and Technology (GIST) Gwangju 500‐712 Republic of Korea; ^5^ Pohang Accelerator Laboratory Pohang University of Science and Technology Pohang 37673 Republic of Korea; ^6^ Division of Advanced Materials Korea Research Institute of Chemical Technology Daejeon 305‐600 Republic of Korea; ^7^ Sensor System Research Center (SSRC) Korea Institute of Science and Technology (KIST) Seoul 02792 Republic of Korea; ^8^ Department of Physics Gyeongsang National University Jinju 52828 Republic of Korea

**Keywords:** AgMn, dual‐phase electrocatalysts, nickel vanadium oxide (NiV_2_O_6_), sequential hydrothermal reaction, Zn‐air batteries

## Abstract

Metal‐air batteries as alternatives to the existing lithium‐ion battery are becoming increasingly attractive sources of power due to their high energy‐cost competitiveness and inherent safety; however, their low oxygen evolution and reduction reaction (OER/ORR) performance and poor operational stability must be overcome prior to commercialization. Herein, it is demonstrated that a novel class of hydrothermally grown dual‐phase heterogeneous electrocatalysts, in which silver‐manganese (AgMn) heterometal nanoparticles are anchored on top of 2D nanosheet‐like nickel vanadium oxide (NiV_2_O_6_), allows an enlarged surface area and efficient charge transfer/redistribution, resulting in a bifunctional OER/ORR superior to those of conventional Pt/C or RuO_2_. The dual‐phase NiV_2_O_6_/AgMn catalysts on the air cathode of a zinc‐air battery lead to a stable discharge–charge voltage gap of 0.83 V at 50 mA cm^−2^, with a specific capacity of 660 mAh g^−1^ and life cycle stabilities of more than 146 h at 10 mA cm^−2^ and 11 h at 50 mA cm^−2^. The proposed new class of dual‐phase NiV_2_O_6_/AgMn catalysts are successfully applied as pouch‐type zinc‐air batteries with long‐term stability over 33.9 h at 10 mA cm^−2^.

## Graphical abstract

The noble dual‐phase heterogeneous electrocatalysts derived by sequential hydrothermal reactions enable an increased surface area and efficient charge transfer/redistribution, resulting in a superior bifunctional oxygen evolution and reduction reaction (OER/ORR). These findings indicate that dual‐phase heterogeneous electrocatalysts may provide a unique opportunity to improve the performance of rechargeable metal‐air battery applications.

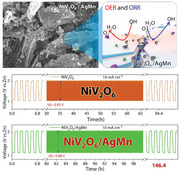

## 1 Introduction

Considerable demand and progress in alternatives to conventional lithium‐ion batteries have led to the aim of commercialization, as such materials would provide an environmentally compatible and sustainable source of electricity.^[^ ^1^ ^]^ Zinc‐air batteries (ZABs) consisting of a nonprecious zinc electrode (anode), alkaline electrolytes, membrane separators, and an air‐permeable carbon sheet (cathode) have been considered the most promising alternative battery technology that could satisfy the above requirements, with a high specific energy density of up to 1218 Wh kg^−1^.^[^ ^2^ ^]^ In addition, ZABs can ensure a flat discharge voltage, safety and environmental benefits, and high volumetric energy density compared to most primary batteries at low cost.^[^ ^3^ ^]^ However, their commercialization has not yet been fully realized because the reaction mechanism of oxygen‐based electrochemistry on the air cathode is sluggish, which causes large overpotentials during the oxygen reduction reaction (ORR) and oxygen evolution reaction (OER) and thereby deteriorates the energy efficiency of ZABs.

Because the slow ORR and OER on the air cathode involve four electron transfer reactions, introducing a precious metal‐based or metal compound‐based bifunctional electrocatalyst (i.e., Ir, Ru, Pt, IrO_2_, and RuO_2_) into the air cathode has been demonstrated to improve oxygen catalysis processes.^[^ ^4^ ^]^ In addition, earth‐abundant catalyst materials, such as iron oxides (FeO), manganese oxides (MnO_2_), cobalt oxides (Co_3_O_4_), and nickel oxides (NiO),^[^ ^5^ ^]^ are also emerging as promising ORR/OER bifunctional electrocatalysts because they are environmentally safe and low cost, and they readily modulate the composition conditions to achieve high catalytic activity for oxygen reactions.^[^ ^6^, ^7^ ^]^ The catalytic efficiency of metal oxides/metals for the ORR/OER can be further improved by employing defect sites, surface/nanostructure modifications and the formation of composite materials.^[^ ^8^ ^]^ However, single‐phase electrocatalysts are not generally sufficient to simultaneously perform both the ORR and OER, resulting in an imbalance of the ORR/OER during charge and discharge processes, respectively, and thus poor operational stability in alkaline electrolytes.^[^ ^9^ ^]^

One desirable approach for securing dual catalytic functions is to develop a dual‐phase heterogeneous electrocatalyst by incorporating ORR‐ and OER‐active elements into advantageous nanostructures.^[^ ^10^ ^]^ For example, promising ORR elements (typically metals such as silver (Ag) and manganese (Mn)) can be physically or chemically bound to OER elements (typically metal oxides such as NiO and Co_3_O_4_).^[^ ^11^ ^]^ A dual‐phase heterogeneous configuration can promote further benefits, such as an increased interfacial area and charge transfer/redistribution, which are not present in conventional single‐phase electrocatalytic nanostructures. Although several studies have been proposed to derive dual‐phase electrocatalytic materials using electrospinning‐calcination processes, wet chemistry and sequential electrodeposition, they still suffer from time‐consuming and rigorous synthetic steps, thus decreasing the reproducibility, cost competitiveness and operational stability.^[^ ^12^ ^]^ Therefore, it has long been desired to establish a simple but efficient binding strategy for ORR and OER elements to derive a new class of dual‐phase heterogeneous electrocatalysts without sacrificing any catalytic efficiency.

Here, we report a new class of hydrothermally grown dual‐phase heterogeneous electrocatalysts consisting of ORR and OER elements that can be used to achieve bifunctional activities and operational stability superior to those of conventional Pt/C or RuO_2_. By employing a hydrothermal synthesis process with high‐purity precursor solutions, AgMn heterometal nanoparticles as ORR elements are homogeneously dispersed on top of a nickel vanadium oxide (NiV_2_O_6_) nanostructure as the OER element to form desirable dual‐phase bifunctional electrocatalysts. The resultant NiV_2_O_6_/AgMn bifunctional electrocatalysts allow an efficient charge transfer/redistribution and increased surface area, leading to a half‐wave potential of 0.83 V for the ORR and an OER overpotential of 1.39 V at *η*_10_ = 10 mA∙cm^−2^. The potential gap, $\Delta E\;(\Delta E=E_{\eta_{10}}-E_{1/2})$, of NiV_2_O_6_/AgMn is 0.56 V. The use of the NiV_2_O_6_/AgMn catalyst as an electrode in a zinc (Zn)‐air battery also shows an excellent discharge–charge voltage gap of 0.83 V at 50 mA cm^−2^, with a specific capacity of 660 mAh g^−1^ and lifecycle stabilities of more than 146 h at 10 mA cm^−2^ and 11 h at 50 mA cm^−2^, thus outperforming the other previously reported dual‐phase catalysts. Furthermore, the NiV_2_O_6_/AgMn catalyst is successfully applied to the realization of a zinc‐air pouch cell with long‐term stability for more than 33.9 h at 10 mA cm^−2^.

## 2 Results and Discussion

### 2.1 Preparation of a NiV_2_O_6_/AgMn Nanostructure via a Sequential Hydrothermal Process

**Figure 1:**
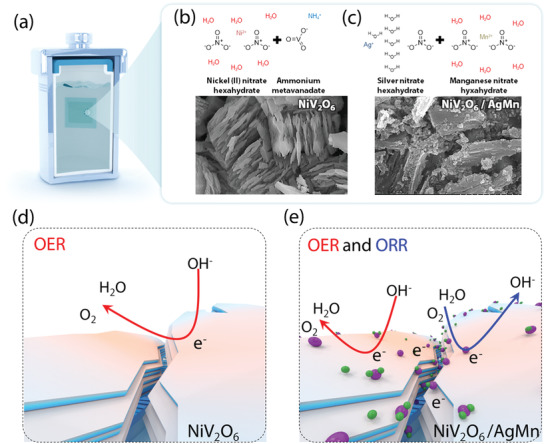
a–c) A schematic representation of facile hydrothermal synthesis with high‐purity metal ion precursors for producing the single‐phase NiV_2_O_6_ 2D nanosheet structure and dual‐phase NiV_2_O_6_/AgMn nanosheets. d,e) Schematic illustration of OER and ORR processes across the single‐phase NiV_2_O_6_ and dual‐phase NiV_2_O_6_/AgMn nanosheet surfaces.

The dual‐phase NiV_2_O_6_/AgMn nanostructure was prepared via a sequential hydrothermal reaction process (**Figure** **1**a). Initially, a high‐purity precursor solution of nickel nitrate hexahydrate (Ni(NO_3_)_2_·6H_2_O) and ammonium metavanadate (NH_4_VO_3_) was heated to 150 °C for 12 h in a sealed autoclave, producing a highly crystalline NiV_2_O_6_ nanostructure on top of a fluorine‐doped tin oxide (FTO) substrate. Then, the subsequent hydrothermal process with silver nitrate hexahydrate (AgH_12_NO_9_) and manganese nitrate hexahydrate (Mn(NO_3_)_2_·6H_2_O) precursor was conducted using the sealed autoclave and NiV_2_O_6_/FTO substrate (for more details, see Experimental Section). Figures S1 and S2, Supporting Information show the XRD patterns of the prepared catalysts. The corresponding high‐resolution scanning electron microscopy (SEM) image of the films after the initial and subsequent hydrothermal processes (Figure 1b,c, respectively) showed that a number of AgMn heterometal nanoparticles were successfully dispersed on top of a distinctive NiV_2_O_6_ 2D sheet‐like structure, indicating the formation of a dual‐phase NiV_2_O_6_/AgMn heterogeneous nanostructure (for more details, see Figures S3–S7,Supporting Information).

In contrast to the single‐phase NiV_2_O_6_ electrocatalyst, which favors the OER process (4OH^−^ → O_2_ + 4e^−^ + 2H_2_O), the dual‐phase NiV_2_O_6_/AgMn heterogeneous nanostructure was hypothesized to be highly beneficial to both the OER and ORR (O_2_ + 2H_2_O + 4e^−^ → 4 OH^−^) processes by i) facilitating fast interfacial electron transfer from AgMn to NiV_2_O_6_, ii) increasing the number of active sites and the surface binding energy, and iii) minimizing the ORR/OER overpotentials required for the abovementioned reactions (Figure 1d,e).

### 2.2 Structural and Morphological Analysis of the NiV_2_O_6_/AgMn Nanostructure

**Figure 2:**
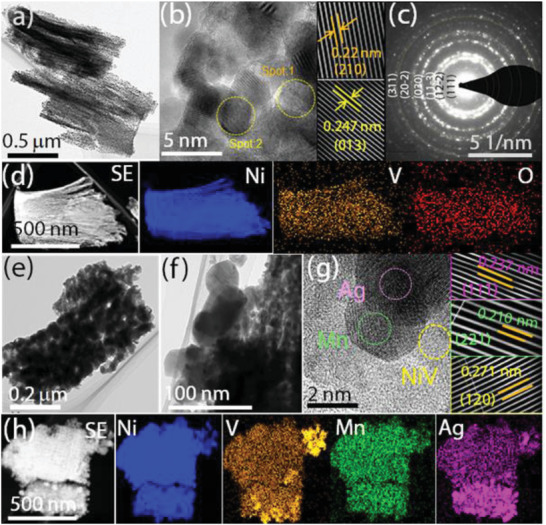
a) Low‐magnification TEM image of NiV_2_O_6_. b) High‐magnification TEM images of nanoparticle‐assembled NiV_2_O_6_ nanosheets and their lattice fringes of NiV_2_O_6_ and c) the corresponding SAED pattern of NiV_2_O_6_. e) STEM image of NiV_2_O_6_ nanosheets and the corresponding elemental color mapping of Ni, V, and O; the scale bar is 500 nm. e) TEM image of the dual‐phase NiV_2_O_6_/AgMn nanosheets. f) TEM images of an individual nanosheet surface encapsulated with AgMn particles. g) High‐magnification TEM image of NiV_2_O_6_/AgMn nanosheets and the corresponding magnified lattice fringes. h) STEM image of NiV_2_O_6_/AgMn and the corresponding elemental color mapping of Ni, V, Ag, Mn, and O by EDX analysis.

To investigate the structure of the dual‐phase NiV_2_O_6_/AgMn obtained by the sequential hydrothermal process, transmission electron microscopy (HR‐TEM) measurements were performed on the single‐phase NiV_2_O_6_ and dual‐phase NiV_2_O_6_/AgMn samples. The TEM image of NiV_2_O_6_ exhibited a 2D nanosheet structure, which is consistent with the SEM analysis described above (**Figure** **2**a). The high‐magnification TEM image of NiV_2_O_6_ clearly indicated a high crystallinity of the primary nanoparticles, and the observed lattice fringes with interplanar distances of 0.220 and 0.247 nm corresponded to the (210) and (013) crystal planes (ICDD card No: 98‐015‐8112) of NiV_2_O_6_ (Figure 2b). The selected area electron diffraction (SAED) ring patterns on NiV_2_O_6_ showed a polycrystalline feature with (111), (12‐2), (11‐3), (030), (20‐2), and (311) facets (ICDD card No: 98‐015‐8112) (Figure 2c). The electron dispersive X‐ray spectroscopy (EDX) measurement on NiV_2_O_6_ provided a vivid elemental color mapping image confirming the surface distributions of Ni, V, and O elements (Figure 2d).

Interestingly, Figure 2e–g show a series of TEM images in which AgMn heterometal nanoparticles are homogeneously anchored on top of the NiV_2_O_6_ nanosheets; the AgMn metal nanoparticles are thought to be precipitated in their metal oxide form, similar to AgMnO*_x_*. The obtained images indicated that the lattice spacing of 0.271 nm was associated with the (120) plane (ICDD card No: 98‐015‐8112) of NiV_2_O_6_, whereas the interplanar spacings of 0.210 and 0.237 nm corresponded to the (221) plane (ICDD card No: 00‐001‐1234) of Mn and the (111) plane (ICDD card No: 00‐001‐1164) of Ag, respectively (see more details in Figures S8 and S9, Supporting Information). Additionally, the TEM images also indicated that the NiV_2_O_6_ spherical particles in the 2D nanosheet structure are strongly interconnected with the AgMn heterometal nanoparticles, implying an increase in the surface area and improvement of charge transport/redistribution during the electrocatalytic process (Figure 2g). The corresponding EDX elemental color mapping distribution showed a uniform distribution of Ag (magenta) and Mn (green) metal nanoparticles across the entire surface of NiV_2_O_6_, confirming successful hydrothermal metal particle decoration on the catalyst surface (Figure 2h). Therefore, the overall SEM and TEM analysis strongly supported the idea that our proposed sequential hydrothermal approach was successfully applied to the formation of dual‐phase NiV_2_O_6_/AgMn heterogeneous nanostructures.

### 2.3 Probing the Electrocatalytic OER and ORR Activity of NiV_2_O_6_/AgMn

**Figure 3:**
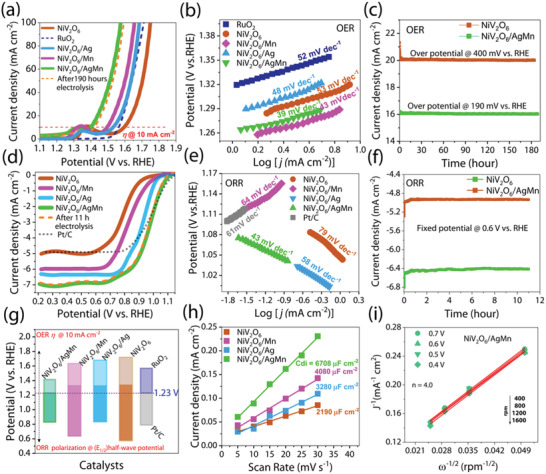
a) Linear sweep voltammetry (LSV) 95% iR corrected polarization curves of commercial RuO_2_, NiV_2_O_6_, NiV_2_O_6_/Ag, NiV_2_O_6_/Mn, and NiV_2_O_6_/AgMn at a scan rate of 5 mV s^−1^ in 1 m KOH. The dotted line (orange) represents the LSV curve of the NiV_2_O_6_/AgMn catalyst performed after the stability test. b) OER Tafel slopes derived from the LSVs of different catalysts. c) Time‐dependent chronoamperometric stability tests of OERs at constant potentials of 1.63 and 1.42 V versus RHE using NiV_2_O_6_ and NiV_2_O_6_/AgMn catalysts as electrodes. d) ORR polarization curves of Pt/C, NiV_2_O_6_, NiV_2_O_6_/Ag, NiV_2_O_6_/Mn, and NiV_2_O_6_/AgMn catalysts at 0.1 m KOH by RDE (1600 rpm). Dashed (orange) ORR curves of NiV_2_O_6_/AgMn obtained after stability tests. e) Tafel slopes obtained from the ORR polarization of different catalysts. f) Current time chronoamperometric durability tests for NiV_2_O_6_ and NiV_2_O_6_/AgMn catalysts in an O_2_‐saturated KOH electrolyte at a constant potential of 0.6 V versus RHE; tests were obtained at 1600 rpm. g) Comparison of OER overpotentials (*η*) obtained at 10 mA cm^−2^ and ORR half‐wave potentials (*E*_1/2_) of various catalysts. h) The calculated electrochemical double‐layer capacitance (*C*_di_) for the catalyst as a function of the scan rate measured in the cyclic voltammetry of the double layer region is a plot showing the *C*_di_ extraction from which the ECSA can be calculated. i) Koutecky–Levich (K–L) plot obtained from the polarization curves at different potentials and corresponding electron transfer numbers (*n*) of NiV_2_O_6_ catalysts in oxygen‐saturated 0.1 m KOH solution.

To clarify the impact of AgMn on the electrocatalytic activity of NiV_2_O_6_, various electrocatalysts, including single‐phase NiV_2_O_6_, dual‐phase NiV_2_O_6_/Ag, NiV_2_O_6_/Mn, and NiV_2_O_6_/AgMn, were evaluated using linear sweep voltammetry (LSV) at a scan rate of 5 mV s^−1^ in a 1 m KOH solution. We found that the single‐phase NiV_2_O_6_ exhibits oxygen evolution overpotentials (*η*_10_) at 360 mV @ 10 mA cm^−1^, which signifies a more positive overpotential than that of the commercial RuO_2_ electrode (*η*_10_ to 330 mA cm^−1^) (**Figure** **3**a). However, significant improvements (295 and 260 mV @ 10 mA cm^−1^) in the OER activity were observed for the dual‐phase NiV_2_O_6_/Ag and NiV_2_O_6_/Mn electrodes compared with those of the single‐phase NiV_2_O_6_. Moreover, the dual‐phase NiV_2_O_6_/AgMn electrode showed a much better OER performance and further reduced overpotential (*η*) at 10 mA cm^−1^ (160 mA), and a 345 mV overpotential was needed to obtain a current density of 100 mA cm^−2^. Notably, the LSV polarization curves (brown dotted lines) for the NiV_2_O_6_/AgMn catalyst after continuous OER electrolysis (189.6 h) exhibited only a slight anodic shift in the potential of 0.019 V versus RHE, indicating that the NiV_2_O_6_/AgMn structures are stable electrocatalysts for the OER. These excellent results mainly originated from a strong electronic interaction^[^ ^12b^ ^]^ between AgMn and NiV_2_O_6_, which tunes the electronic structure of the catalyst to facilitate Ni^2+^ to Ni^3+^ or Ni^4+[^ ^13^, ^14^, ^15^, ^16^, ^17^ ^]^ (Figure S10, Supporting Information). The amount of O_2_ generated during the OER process of the NiV_2_O_6_/AgMn electrocatalysts was assessed using a gas chromatographic technique. Chronopotentiometry was performed in 1.0 m KOH with a constant current density of 10 mA cm^−2^ at 5 h. The time dependence of oxygen evolution in NiV_2_O_6_/AgMn catalysts (Figure S11, Supporting Information) showed that the Faradaic efficiency for the OER reached nearly 100%.

The reaction kinetics of the OER process in various electrocatalysts, including conventional RuO_2_, NiV_2_O_6_, NiV_2_O_6_/Ag, NiV_2_O_6_/Mn, and NiV_2_O_6_/AgMn, were also evaluated using Tafel analysis (Figure 3b). The Tafel slopes of the above materials were calculated to be 52.1, 53.2, 48.4, 43.2, and 39.1 mV dec^−1^, respectively. Among all of the samples, the NiV_2_O_6_/AgMn catalyst exhibited the fastest kinetics and the smallest Tafel slope, implying that AgMn anchored on top of NiV_2_O_6_ can modulate the catalytic activity by tuning the intrinsic kinetics. The turnover frequencies (TOFs) of NiV_2_O_6_/AgMn for the OER are superior to those of the individual NiV_2_O_6_, NiV_2_O_6_/Mn, and NiV_2_O_6_/Ag catalysts. At an overpotential of 320 mV versus RHE (details of the calculation are given in the Supporting Information), the TOF of NiV_2_O_6_/AgMn was calculated to be 0.344 s^−1^, which was 28‐fold, 53‐fold, and 138‐fold those for the OER on NiV_2_O_6_/Mn (0.0123 s^−1^), NiV_2_O_6_/Ag (0.0065 s^−1^), and NiV_2_O_6_ (0.0025 s^−1^), respectively. The higher TOF value of NiV_2_O_6_/AgMn is consistent with the improved OER process rate. In addition, the electrochemical operational durability of the OER was assessed using chronoamperometric techniques at an overpotential of 400 mV versus RHE (1 m KOH) for NiV_2_O_6_ (≈185 h) and 190 mV versus RHE (1 m KOH) for NiV_2_O_6_/AgMn for 189.6 h, resulting in reductions in the current density of 13% and 5%, respectively (Figure 3c).

In parallel, the ORR electrocatalytic activity of the NiV_2_O_6_, NiV_2_O_6_/Ag, NiV_2_O_6_/Mn, NiV_2_O_6_/AgMn catalysts was evaluated using a rotating disc electrode (RDE) at 1600 rpm in an O_2_‐saturated 0.1 m KOH electrolyte (Figure 3d). The optimized NiV_2_O_6_/AgMn catalysts showed a more positive shift of the onset potential of *E*_0_ = 1.10 V versus RHE, which is higher than those of NiV_2_O_6_/Ag (*E*_0_ = 1.05 V RHE), NiV_2_O_6_/Mn (*E*_0_ = 1.01 V RHE), NiV_2_O_6_ (*E*_0_ = 0.98 V RHE) and the commercial Pt/C electrode (*E*_0_ = 1.10 V RHE) in an alkaline medium. The ORR half‐wave potential (*E*_1/2_) shift also has a trend similar to that of the onset potential, following the order of NiV_2_O_6_/AgMn (0.83 V) > NiV_2_O_6_/Ag (0.79 V) > NiV_2_O_6_/Mn > (0.62 V) > NiV_2_O_6_ (0.55 V). The NiV_2_O_6_/AgMn catalysts exhibit a larger diffusion‐limited current density of −6.94 mA cm^−2^ than NiV_2_O_6_/Ag (−6.29 mA cm^−2^), NiV_2_O_6_/Mn (−5.98 mA cm^−2^), and the parent NiV_2_O_6_ (−4.94 mA cm^−2^) compared with those of the commercial Pt/C electrode (−4.93 mA cm^−2^). The reaction kinetics in the ORR process were also investigated by Tafel plots. The NiV_2_O_6_/AgMn (43 mV dec^−1^) catalyst displayed a smaller Tafel slope than the NiV_2_O_6_/Ag (58 mV dec^−1^), NiV_2_O_6_/Mn (64 mV dec^−1^), NiV_2_O_6_ (79 mV dec^−1^), and Pt/C (61 mV dec^−1^) catalysts, revealing fast reaction kinetics during the ORR process (Figure 3e).

The operational stability of NiV_2_O_6_ and NiV_2_O_6_/AgMn during the ORR process was evaluated by a continuous chronoamperometric method in a 0.1 m KOH electrolyte at a constant potential of 0.6 V versus RHE with a rotating speed at 1600 rpm for 11 h (Figure 3f). The stable current density for the NiV_2_O_6_ and NiV_2_O_6_/AgMn catalysts even after 11 h indicated promising operational stability. Furthermore, after continuous ORR electrolysis, the optimized NiV_2_O_6_/AgMn catalyst was confirmed again by LSV under the same initial measurement conditions, leading to an acceptable cathodic shift potential of 0.008 V versus RHE, thereby suggesting the good stability of the dual‐phase NiV_2_O_6_/AgMn nanosheet structure toward the ORR process in an alkaline electrolyte (Figure 3d, brown dashed line).

The overall bifunctional electrocatalytic activity of NiV_2_O_6_/AgMn was estimated from the potential difference (Δ*E*) (Δ*E* = *E_η_* − *E*_1/2_) between the OER overpotential (*η_j_* = _10_) measured at a current density of 10 mA cm^−2^ and the ORR half‐wave potential (*E*_1/2_) (Figure 3g). The $\Delta E\;(\Delta E=E_{\eta_{10}}-E_{1/2})$ of NiV_2_O_6_/AgMn was 0.56 V, which is much lower than those of NiV_2_O_6_/Ag, NiV_2_O_6_/Mn, NiV_2_O_6_ and previously reported electrocatalysts (see more details in Figure S12 and Table S1, Supporting Information).^[^ ^18^, ^19^, ^20^, ^21^, ^22^ ^]^ Note that AgMn electrocatalysts exhibited a much higher OER/ORR performance than those of pure Ag or Mn electrocatalysts; these outstanding features of AgMn could be related to i) the increase in the surface area (or number of active sites) and ii) the fast interfacial electron transfer from AgMn to NiV_2_O_6_ (Figure S13, Supporting Information). To explore the active surface areas of the catalysts, we calculated the electrochemically active surface area (ECSA) of each electrode using cyclic voltammetry (CV) with varying scan rates (mV s^−1^) in the non‐Faradic potential region (Figure S14, Supporting Information). Figure 3h shows that NiV_2_O_6_/AgMn has the highest *C*_dI_ value of 6708 µF cm^−2^ among the NiV_2_O_6_/Ag, NiV_2_O_6_/Mn, and NiV_2_O_6_ catalysts. Moreover, the NiV_2_O_6_/AgMn catalyst had the highest ECSA of 168.0 cm^2^ and a large surface roughness, while the ECSA values of NiV_2_O_6_/Mn, NiV_2_O_6_/Ag, and NiV_2_O_6_ were measured to be 102.8, 82.8, and 54.7 cm^2^, respectively (Table S2, Supporting Information). The electron transfer number *n* value in the ORR process on NiV_2_O_6_/AgMn was also estimated by the ORR polarization curves with different rotation speeds (600–1800 rpm) and the Koutecky–Levich equation. The K–L plots for NiV_2_O_6_ and NiV_2_O_6_/AgMn show first‐order kinetics over a range of potentials at different rotational rates, resulting in calculated electron transfer numbers (*n*) of 3.9 and 4, respectively (Figure 3i and Figure S15 and Table S3, Supporting Information). This indicated that the ORR process has strong selectivity for the four‐electron reduction from O_2_ to H_2_O for the NiV_2_O_6_/AgMn surfaces.

### 2.4 Chemical Interactions Among the Components of NiV_2_O_6_/AgMn

**Figure 4:**
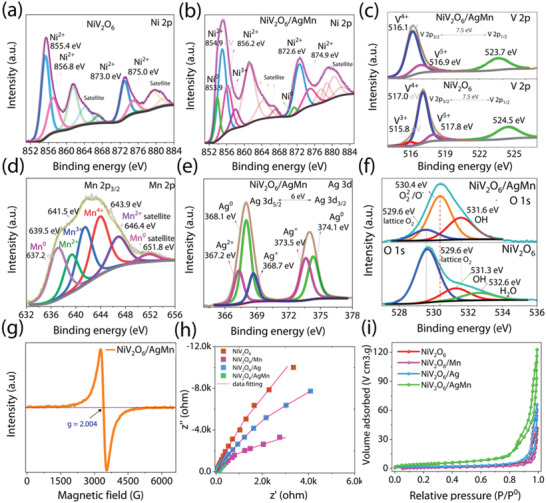
X‐ray photoelectron spectroscopy (XPS) analysis of the NiV_2_O_6_ and NiV_2_O_6_/AgMn catalysts. High resolution Ni 2p spectra of a) NiV_2_O_6_ and b) NiV_2_O_6_/AgMn catalysts, c) high‐resolution V 2p spectra of NiV_2_O_6_ and NiV_2_O_6_/AgMn catalysts, d) high resolution Mn 2p spectrum of NiV_2_O_6_/AgMn catalyst, e) high‐resolution Ag 3d spectrum of NiV_2_O_6_/AgMn catalyst, f) O 1s spectra of NiV_2_O_6_‐ and NiV_2_O_6_/AgMn catalysts, g) electron paramagnetic resonance spectrum of NiV_2_O_6_/AgMn catalyst, h) electrochemical impedance (EIS) spectra of NiV_2_O_6_, NiV_2_O_6_/Ag, NiV_2_O_6_/Mn, and NiV_2_O_6_/AgMn catalysts recorded at an open circuit potential, and i) nitrogen adsorption/desorption isotherms of NiV_2_O_6_, NiV_2_O_6_/Ag, NiV_2_O_6_/Mn, and NiV_2_O_6_/AgMn electrocatalysts.

To understand the origin of the excellent dual catalytic activity of NiV_2_O_6_/AgMn, we performed X‐ray photoemission spectroscopy (XPS) to analyze the electronic interactions of the elements on the catalyst surface (see XPS survey and C 1s XPS spectra of NiV_2_O_6_ and NiV_2_O_6_/AgMn catalysts in Figures S16 and S17, Supporting Information). For the high‐resolution NiV_2_O_6_ spectra, the Ni 2p peaks with binding energies of 855.4/873.0 eV and 856.8/875.0 eV corresponded to Ni 2p_3/2_ and Ni 2p_1/2_, respectively, indicating the presence of Ni^2+^ because of NiO and Ni(OH)_2_, respectively (**Figure** **4**a). However, the Ni 2p binding energies of NiO (854.9/872.6 eV)^[^ ^23^, ^24^ ^]^ and Ni(OH)_2_ (856.2/874.9 eV)^[^ ^25^ ^]^ in NiV_2_O_6_/AgMn were negatively shifted compared to that of pristine NiV_2_O_6_ (Figure 4b)_._ Interestingly, after a second annealing process of NiV_2_O_6_/AgMn at 400 °C, two distinct metal peaks of Ni were detected at 853.9 (Ni 2p_3/2_) and 871.3 eV (Ni 2p_1/2_).^[^ ^26^ ^]^ In addition, the Ni 2p core level XPS spectrum in Figure 4b shows a new peak at 858.0 eV,^[^ ^20^, ^27^, ^28^, ^29^, ^30^, ^31^ ^]^ suggesting the formation of Ni^3+^ on the NiV_2_O_6_/AgMn surface after AgMn decoration. The oxidation peak associated with Ni^3+^ corresponds to an active site for the OER, and consequently, the enhanced Ni^3+^ oxidation state provides sufficient bond strength for intermediate reactions at the catalyst surface, leading to optimized OER performance.^[^ ^32^, ^33^, ^34^ ^]^

In the high‐resolution V 2p spectrum, the main peak binding energies of 517.0/524.5 eV corresponding to the V 2p_3/2_ and V 2p_1/2_ spin orbitals were attributed to V^4+^, as shown in Figure 4c. The binding energies of the V 2p_3/2_ spin orbital peaks of 515.8 and 517.8 eV were also associated with V^3+^ and V^5+^, respectively.^[^ ^35^, ^36^ ^]^ However, for NiV_2_O_6_/AgMn, the peak of V^5+^ (516.9 eV) and the peak of V^4+^ (516.1 and 523.7 eV)^[^ ^37^ ^]^ shifted to lower binding energies, implying an electronic connection with adjacent metal particles. Indeed, we found that the Mn 2p core level of NiV_2_O_6_/AgMn showed distinct metallic Mn^0^ peaks at 637.2 and 651.8 eV for the Mn 2p_3/2_ spin orbital due to the reduction of some of the MnO*_x_* to the metallic state Mn^0^ (Figure 4d)^[^ ^38^ ^]^ (a detailed view of the XPS spectrum of the AgMn nanoparticles is shown in Figure S18, Supporting Information). For the core‐level Ag spectrum, two distinct metal (Ag^0^) peaks corresponding to Ag 3d_5/2_ and Ag 3d_3/2_ and a splitting energy of 6 eV for NiV_2_O_6_/AgMn were located at 368.1 and 374.1 eV^[^ ^39^ ^]^ (Figure 4e). Additional fitting peaks for the Ag 3d_5/2_ and Ag 3d_3/2_ spin orbitals at 367.2, 373.5, and 368.7 eV corresponded to the Ag^2+^ and Ag^1+^ states, respectively.^[^ ^40^, ^41^ ^]^

Figure 4f shows the O 1s core level XPS spectra of both NiV_2_O_6_ and NiV_2_O_6_/AgMn fitted with four typical characteristic peaks: metal lattice oxygen species (529.6 eV for O^2−^), highly oxidative oxygen species (530.6 eV for O_2_^2−^, O^−^), hydroxyl species or surface adsorbed oxygen (531.6 eV for OH or O_2_) and adsorbed water molecules (532.6 eV for H_2_O).^[^ ^42^, ^43^, ^44^, ^45^ ^]^ The relative amounts of the four oxygen species in the NiV_2_O_6_ and NiV_2_O_6_/AgMn catalysts were calculated as the sum of the relative areas of the various peaks, and the results are listed in Table S4, Supporting Information. The relative amount of 35.89% highly oxidative oxygen species (surface peroxides O_2_^2−^ and O^−^) on the NiV_2_O_6_/AgMn catalyst surface has been reported to strongly correlate with the concentration of surface oxygen species contributing to superior ORR and OER activity.^[^ ^45^, ^46^, ^47^, ^48^, ^49^ ^]^

Furthermore, the negative shift of the Ni and V binding energies of NiV_2_O_6_/AgMn suggests rapid electron transfer from AgMn metal particles to NiV_2_O_6_ and an increase in the electron density around NiV_2_O_6_. These core‐level shifts are substantially more sensitive to the modulated electronic properties of NiV_2_O_6_/AgMn, which can result in partial loss of oxygen in the NiV_2_O_6_ lattice, resulting in oxygen vacancies.^[^ ^50^ ^]^ The presence of oxygen vacancies (OVs) was confirmed by a strong signal at *g* = 2.004 in the electron paramagnetic resonance (EPR) spectrum, which was strongly correlated with the presence of unpaired electrons^[^ ^51^, ^52^, ^53^ ^]^ (Figure 4g). Therefore, the presence of AgMn metal and oxygen vacancies was confirmed in NiV_2_O_6_/AgMn, which is very beneficial for tuning the surface catalytic activity and electronic properties and thus enhancing their OER/ORR activity.

In addition, the confirmed AgMn metal and oxygen vacancies generated an excess electron density in the catalyst to enhance electrical conductivity and improve charge transfer during electrolysis of the OER and ORR.^[^ ^54^, ^55^, ^56^, ^57^ ^]^ This conclusion was further substantiated by Nyquist plot analysis. Figure 4h shows the Nyquist plots obtained from fitting the electrochemical impedance spectroscopy (EIS) results of the NiV_2_O_6_, NiV_2_O_6_/Ag, NiV_2_O_6_/Mn, and NiV_2_O_6_/AgMn samples using the simplified Randles equivalent circuit (Figure S19 and Table S5, Supporting Information). The charge transfer resistance (*R*_ct_) values for NiV_2_O_6_, NiV_2_O_6_/Ag, NiV_2_O_6_/Mn, and NiV_2_O_6_/AgMn were 6996, 4300, 1172, and 539, respectively, exhibiting the lower internal resistance and faster charge transfer ability of NiV_2_O_6_/AgMn. Furthermore, type III N_2_ isotherm curves with H_3_ hysteresis loops were also observed for all of the catalysts, indicating the presence of pores in the samples (Figure 4i and Figure S20a, Supporting Information). The Barrett–Joyner–Halenda pore size distribution curve of the sample shows that the mesopore pore size diameter was 3–50 nm (Figure S20b and Table S6, Supporting Information). The specific surface area of NiV_2_O_6_/AgMn was 196 m^2^ g^−1^, which was more than twice that of NiV_2_O_6_ (c.f., 90, 120, and 135 m^2^ g^−1^ for NiV_2_O_6_, NiV_2_O_6_/Ag, and NiV_2_O_6_/Mn, respectively).

### 2.5 Understanding the Phenomenon of the Presence of AgMn Metal and Oxygen Vacancies Using DFT Calculations

**Figure 5:**
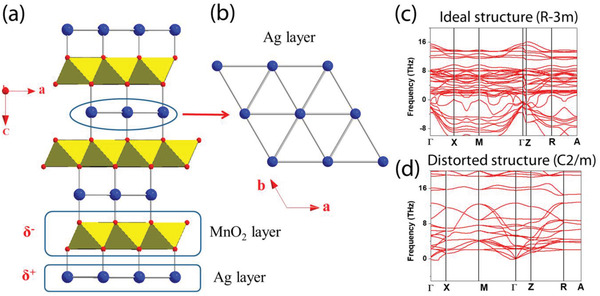
a) Perspective view of the AgMnO_2_ crystal structure and b) Ag triangular net layer; the yellow polyhedral, blue circles, and red circles refer to MnO_6_ octahedra, Ag atoms, and O atoms. c,d) Simulated phonon band dispersion relations for ideal (R‐3m) and distorted (C2/m) AgMnO_2_ structures, respectively.

To clarify the reason for the presence of AgMn metal and the formation of oxygen vacancies on top of NiV_2_O_6_, we performed calculations based on density functional theory (DFT) with a simple crystal structure of AgMnO_2,_ which has a trigonal symmetry [SG(space group): R‐3m] (R1), as revealed by grazing incident wide‐angle X‐ray scattering (GIWAXS) measurements (see Figure S21 and Table S7, Supporting Information). **Figure** **5**a shows that Ag^+^ (d^10^) forms a triangular net in the ab plane and that Mn^3+^ (d^4^) forms a MnO_2_ layer by sharing the edge of MnO_6_ octahedra in the ab plane, while the MnO_2_ layer is sandwiched between Ag triangular nets along the c direction. For the Ag triangular net of AgMnO_2_, the Ag^+^ ions interact with the nearest O^2−^ of the MnO_2_ layer along the c direction (Figure 5b). The interaction between Ag^+^ (d^10^) ions causes instability in the AgMnO_2_ system because the fully occupied antibonding level increases their instability. To relieve this instability, AgMnO_2_ undergoes a phase transition by distorting its structure or developing Ag vacancies. For this reason, the experimental Ag‐deficient Ag_0.9_MnO_2_ structure is distorted from the ideal R‐3m structure, inducing crystallization of monoclinic C2/m. Indeed, a negative frequency indicating the dynamical instability of the structure was observed in the R‐3m structure, while the negative frequency disappeared in the monoclinic C2/m structure, implying that there is a possibility of developing Ag vacancies to stabilize the system (see the calculated phonon dispersion relation both before and after the structural phase transition in Figure 5c,d, respectively).

Based on the analysis above, one could imagine that the increase in the Ag vacancy concentration increases the degree of structural strain in the system, while the presence of Ag vacancies might cause the development of Mn vacancies in AgMnO_2_ to release the structural strain. To evaluate this hypothesis, we determined the Mn‐vacancy formation energy for pure AgMnO_2_ and Ag vacancy‐developed AgMnO_2_ (Ag_1‐x_MnO_2_) via a DFT approach. The calculated Mn vacancy formation energies were 196 meV/FU and −63 meV for pure AgMnO_2_ and Ag_1−_ *_x_* MnO_2_, respectively, which implied that the Mn vacancy would be developed by increasing the Ag vacancy concentration. Therefore, it could be reasonable to conclude that the presence of Ag vacancies and Mn vacancies in AgMnO_2_ leads to the precipitation of AgMn metal clusters, which is in good agreement with the experimental observations presented in Figure 2.

Note that the charge carrier confined in the MnO_2_ layer in AgMnO_2_ would lead to the development of oxygen vacancies caused by electron enrichment in the MnO_2_ layer. Moreover, the formation of oxygen vacancies compensates for the charge imbalance in vacancies developed Ag_1−_ *_x_* Mn_1−_ *_y_* O_2_ (Figure S22, Supporting Information).

### 2.6 NiV_2_O_6_/AgMn as an Air Cathode for a Highly Efficient and Stable Zn‐Air Battery

The technical impact of the NiV_2_O_6_/AgMn catalyst on the dual‐function activity and stability in the electrochemical reaction was proven by a homemade rechargeable Zn‐air battery. The NiV_2_O_6_/AgMn Zn‐air battery was assembled with a Zn plate, and NiV_2_O_6_/AgMn catalyst‐coated carbon cloth was used as the anode and cathode components, respectively. The NiV_2_O_6_ catalyst was also assembled with ZAB, and the power and discharge energy densities of NiV_2_O_6_/Mn and NiV_2_O_6_/Ag samples were also evaluated in 6 m KOH containing 0.2 m zinc acetate (Zn C_4_H_6_O_4_) electrolyte solution for comparison.

**Figure 6:**
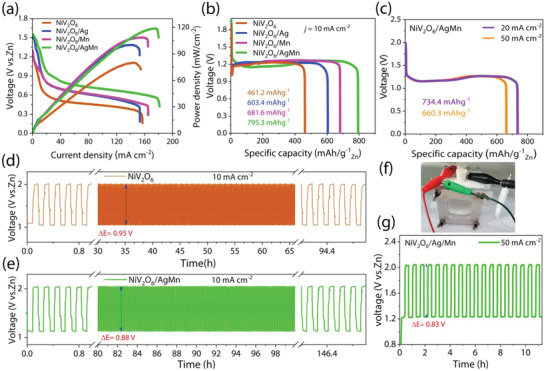
a) Discharge polarization plots and the corresponding power density curves for NiV_2_O_6_, NiV_2_O_6_/Ag, NiV_2_O_6_/Mn, and NiV_2_O_6_/AgMn catalyst‐based Zn‐air battery cells. b) Galvanostatic voltage‐specific capacity discharge curves of Zn‐air battery cells with different catalysts when discharged at 10 mA cm^−2^. c) NiV_2_O_6_/AgMn catalyst‐based Zn‐air battery cell when discharged at 20 and 50 mA cm^−2^. d,e) Comparison of the galvanostatic discharge‒charge voltage cycling profiles for NiV_2_O_6_ and NiV_2_O_6_/AgMn catalyst‐based Zn‐air battery cells at 10 mA cm^−2^. f) A photograph of a two‐electrode liquid homemade rechargeable battery. g) Discharge–charge voltage cycling stabilities of the NiV_2_O_6_/AgMn catalyst‐based Zn‐air battery cell at 50 mA cm^−2^.

**Figure** **6**a shows the polarization (*I–V*) curves and corresponding power density (*P*–*V*) plots of Zn‐air battery cells based on NiV_2_O_6_, NiV_2_O_6_/Ag, NiV_2_O_6_/Mn, and NiV_2_O_6_/AgMn catalysts. The NiV_2_O_6_/AgMn Zn‐air battery cell exhibited a power density of 119 mW cm^−2^ at 180 mA cm^−2^, which is significantly higher than the density of 108 mW cm^−2^ for the NiV_2_O_6_/Mn‐based Zn‐air battery cell at 164 mA cm^−2^, and it also far exceeds those of the NiV_2_O_6_/Ag‐based Zn‐air battery (100 mW cm^−2^ at 152 mA cm^−2^) and the NiV_2_O_6‐_based Zn‐air battery (80 mW cm^−2^ obtained at 154 mA cm^−2^). The open circuit voltages (OCVs) of Zn‐air batteries with NiV_2_O_6_/AgMn, NiV_2_O_6_/Mn, NiV_2_O_6_/Ag, and NiV_2_O_6_ catalysts are ≈1.53, ≈1.36, ≈1.51, and ≈1.34 V, respectively, as determined from the polarization profiles.

Galvanostatic discharge polarization curves are shown in Figure 6b. When the current density was 10 mA cm^−2^, the Zn‐air battery catalyzed by the NiV_2_O_6_/AgMn sample delivered a specific capacity of 795.3 mAh g^−1^, surpassing those of the NiV_2_O_6_/Mn‐, NiV_2_O_6_/Ag‐, and NiV_2_O_6_‐based Zn‐air cells, which were 681.6, 603.4, and 461.2 mAh g^−1^, respectively. Notably, the zinc‐air battery catalyst with the NiV_2_O_6_/AgMn sample can withstand high current densities of 20 and 50 mA cm^−2^ and provide specific capacities of 734.4 and 660 mAh g^−1^, respectively (Figure 6c).

Charge‒discharge cycle tests of NiV_2_O_6_‐ and NiV_2_O_6_/AgMn‐catalyzed Zn‐air batteries were also performed by galvanostatic measurement conducted with 20 min cycles. In each cycle, the cell was charged for 10 min and then discharged for 10 min. At room temperature and a constant current density of 10 mA cm^−2^, the NiV_2_O_6_/AgMn‐catalyzed Zn‐air battery displayed cyclic discharge and charge potentials of 1.14 and 2.02 V, corresponding to a small voltage gap (Δ*V*) of 0.88 V. In addition, the superior performance of NiV_2_O_6_/AgMn‐catalyzed Zn‐air batteries was advantageous compared to that of NiV_2_O_6_‐catalyzed Zn‐air batteries, with discharge and charge potentials of 1.06 and 2.01 V, leading to a voltage gap (Δ*V*) of 0.95 V. Therefore, the lower charge–discharge voltage gap and stable performance with rechargability obtained for 146.8 h (as shown in Figure 6d,e) in the Zn‐air battery with the NiV_2_O_6_/AgMn catalysts were much better than those in the NiV_2_O_6_‐catalyzed zinc‐air battery (94.8 h). The cycling performance of the catalyst was evaluated using the Zn‐air battery cell shown in Figure 6f. We further tested the stability of NiV_2_O_6_/AgMn, as shown in Figure 6g. The individual cycle involved charging for 30 min and then discharging for 30 min, and the performance of the Zn‐air battery with the NiV_2_O_6_/AgMn catalyst remained almost unchanged (Δ*V* = 0.83 V) for more than 11 h, even at a high current density of 50 mA cm^−2^, demonstrating that the NiV_2_O_6_/AgMn structure is stable and practical.

**Figure 7:**
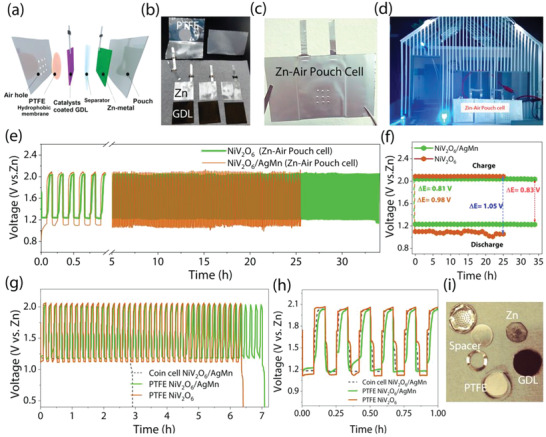
a) Schematic diagram of the fabrication methods for the Zn‐air full battery assembled using NiV_2_O_6_/AgMn catalyst loaded with a GDL as the cathode and a commercial Zn plate as the anode. b,c) Photograph description of a Zn‐air full battery fabricated and assembled using NiV_2_O_6_/AgMn as the catalyst. d) Photograph showing blue and white LED light powered by the assembled Zn‐air batteries based on the NiV_2_O_6_/AgMn catalyst in series. e,f) Discharge‒charge voltage cycling profiles of NiV_2_O_6_ and NiV_2_O_6_/AgMn zinc‐air batteries at 10 mA cm^−2^. g,h) The discharge–charge voltage cycling stabilities of the coin cell. i) Pictures of NiV_2_O_6_/AgMn catalyst‐based Zn‐air coin cells after analysis.

Moreover, we designed a special structure in the form of pouch cells to drive zinc‐air batteries. A polypropylene‐coated aluminum pouch was used as a package, and a pinhole was drilled at the catalyst reaction side to allow air to enter and exit. In addition, a polytetrafluoroethylene (PTFE) film was attached to prevent leakage and evaporation of the electrolyte through the pinhole so that it could be operated for a long time. A catalyst‐coated gas diffusion layer (GDL), glass fiber separator, and zinc metal plate were prepared inside, and aluminum lead tabs were attached to each electrode. After setting the interiors, both sides were sealed by a thermal press machine. Finally, a zinc‐air battery was completed by injecting electrolyte into the pouch cell and sealing it. Photographs of the NiV_2_O_6_/AgMn catalyst‐based rechargeable Zn‐air battery pouch cell are shown in **Figure** **7**a–c. A demonstration of lighting an LED house continuously for 33 h was also conducted by connecting three prepared pouch cells (OCV of 4.22 V, while a single battery is 1.40 V) in series to match the voltage (Figure 7d). To connect each cell, ultrasonic welding was used to minimize the electrical resistance. As shown in Figure 7e,f, the Zn‐air battery pouch cell catalyzed by NiV_2_O_6_/AgMn shows a small charge‒discharge voltage gap of 0.81 V, and after 33.9 h of continuous operation, there was no obvious voltage drop (Δ*V* = 0.808 V), indicating the excellent durability. In contrast, the Zn‐air battery pouch cell catalyzed by NiV_2_O_6_ presented limited stability of only 25 h, as seen in (Δ*V* 0.98 V initial to 1.023 V final). For comparison, NiV_2_O_6_ and NiV_2_O_6_/AgMn catalysts with Zn‐air coin cells were also constructed with and without PTFE film (Figure 7g–i). Zn‐air batteries with NiV_2_O_6_/AgMn catalysts and PTFE film exhibited very stable cyclic discharging and charging for 7.1 h with a smaller voltage gap of 0.87 V than Zn‐air batteries with the NiV_2_O_6_ catalyst (6.4 h, Δ*V* = 0.96 V). These results further reveal that NiV_2_O_6_/AgMn is a potential candidate for efficient rechargeable Zn‐air battery applications.

## 3 Conclusion

In conclusion, we demonstrated that the proposed new class of dual‐phase NiV_2_O_6_/AgMn heterogeneous electrocatalysts can be obtained by a sequential hydrothermal method. The strong electronic interactions between highly active NiV_2_O_6_ and AgMn catalysts resulted in superior bifunctional electrocatalytic activity for both the ORR and the OER compared to conventional Pt/C or RuO_2_. By employing TEM and advanced electrochemical analysis methods, we confirmed that the excellent bifunctional electrocatalytic activity of NiV_2_O_6_/AgMn was attributed to the synergistic interplay of NiV_2_O_6_ with abundant Ag and Mn metal active centers, which can accelerate the catalytic activity due to the increase in active surface sites. In addition, we found that the formation of oxygen vacancies in AgMnO_2_ and the favorable electronic structure of NiV_2_O_6_/AgMn not only lowered the charge transfer resistance but also provided a fast charge transfer rate in the ORR/OER process. The formation of the active intermediate Ni^3+^ and highly oxidative oxygen species (O_2_^2−^/O^−^) on the NiV_2_O_6_/AgMn surface also contributed to the additional catalytic support to further enhance the bifunctional activity. The derived NiV_2_O_6_/AgMn catalyst was used as an air cathode for highly efficient and stable Zn‐air batteries, resulting in an excellent discharge–charge voltage gap of 0.83 V at 50 mA cm^−2^, with a specific capacity of 660 mAh g^−1^ and life cycle stabilities of more than 146 h at 10 mAcm^−2^ and 11 h at 50 mA cm^−2^. Thus, the NiV_2_O_6_/AgMn catalyst was successfully applied to the practical application of zinc‐air pouch cells with long stability of over 33.9 h at 10 mA cm^−2^. These results provide an example of the successful combination of dual‐phase catalysts as a new class of ORR/OER bifunctional electrocatalysts and the prospect of practical applications in the field of Zn‐air batteries and energy conversion.

## 4 Experimental Section

### Synthesis of NiV_2_O_6_ Nanosheets

NiV_2_O_6_ nanosheets were prepared on FTO substrates by a hydrothermal method. Before preparing the NiV_2_O_6_ nanosheets, the FTO‐coated glass surface (2 cm × 2 cm) was cleaned sequentially with acetone, ethanol, and deionized water in an ultrasonic bath for 10 min each to remove surface impurities. To fabricate the NiV_2_O_6_ microsheets, a seed layer of NiV particles was coated on the substrate. Then, 0.01 m nickel nitrate hexahydrate Ni(NO_3_)_2_·6H_2_O and 0.01 m ammonium metavanadate NH_4_VO_3_ (Sigma‐Aldrich, 97%) were mixed with 50 mL of isopropanol and stirred at 45 °C for 35 min to form a homogeneous solution. The FTO substrate was vertically immersed in the solution for 5 min. After rinsing with a mixture of ethanol and deionized water (1:1), the samples were dried at 100 °C under air for 30 min. To prepare NiV_2_O_6_ nanosheets in FTO, 0.01 mol of Ni(NO_3_)_2_·6H_2_O and 0.01 mol of NH_4_VO_3_ together with 50 mL of 0.02 mol of aqueous citric acid solution were vigorously stirred until a homogeneous solution was formed. ≈18 mL of an ethylene glycol (EG) dispersant, 2.6 mmol of a polyvinyl alcohol polymerization agent, and 0.85 mmol of a cetyltrimethylammonium bromide (CTAB)‐based surfactant were added to the solution, followed by further stirring at 35 °C for 2 h. The mixed solution was then poured into a Teflon‐lined autoclave containing a NiV seed layer‐coated FTO plate, sealed in a stainless steel tank, and heated at 120 °C for 10 h. After the hydrothermal reaction, the samples were collected and washed with a mixed solvent of deionized water and ethanol (1:1 v/v ratio) to remove residual ions and excess surfactant. The resulting final sample was calcined in a muffle furnace at a heating rate of 5 °C min^−1^ for 2 h at 500 °C to successfully obtain mesoporous hierarchical NiV_2_O_6_ nanosheets.

### Synthesis of AgMn Heterometal Nanoparticles

Ag/Mn metal nanoparticles were prepared by reacting stoichiometric amounts of the two metal precursors. Briefly, 18 mmol of manganese(II) nitrate hexahydrate Mn(NO_3_)_2_·6H_2_O was mixed in 50 mL (8.4 g, 40 mmol) of weak triprotic citric acid. After that, 20 mg of a mild reducing agent, polyvinylpyrrolidone (2.0 µmol) metal stabilizer, 12 mmol EG (a dispersant and reducing agent), and 2 mmol of a CTAB surfactant were added to the reaction solution, and the mixture was stirred for 1 h to obtain a homogeneous solution. Then, 5 mL of sodium borohydride (NaBH_4_) and 16 mmol of silver nitrate hexahydrate (AgNO_3_) source mixture were added to the solution, and stirring was continued in a reflux condenser at 40 °C for 30 min to obtain a homogeneous solution. The resulting solution was transferred to a Teflon‐lined autoclave and heated at 150 °C for 1 h. After the hydrothermal reaction was completed, the AgMn metal precursor was recovered from the liquid by centrifugation, followed by repeated washing with acetone. The resulting precursor was heat treated at 400 °C for 2 h (5 °C min^−1^) to remove the remaining organic material and to obtain AgMn heterometal nanoparticles. For comparison, Ag‐ and Mn‐only metal nanoparticles were synthesized in a similar manner, except Mn and Ag were used as precursors.

### Decoration of AgMn Heterometal Nanoparticles over the NiV_2_O_6_ Nanosheets

To decorate the NiV_2_O_6_ surface with AgMn metal nanoparticles, AgMn metal particles were directly deposited on the NiV_2_O_6_ surface by a hydrothermal method. 30 mg of AgMn metal nanoparticles was continuously sonicated in 50 mL of acetyl acetone nanoparticle capping agent for 10 min to ensure uniform dispersion. Then, the AgMn metal nanoparticle dispersion was transferred to a Teflon‐lined autoclave containing the prepared NiV_2_O_6_ nanosheets, sealed in a stainless steel tank, placed in an electric oven, and heated at 120 °C for 1 h. The collected sample was heat treated at 400 °C for 1 h to obtain a final NiV_2_O_6_/AgMn electrode material.

### Characterizations of Dual‐Phase NiV_2_O_6_/AgMn

X‐ray diffraction analysis was performed on an X'Pert PRO operated at 40 kV using Cu K*α* radiation (*λ* = 1.5406 Å). The nitrogen adsorption and desorption isotherms of the prepared samples were investigated with a Micromeritics ASAP 2020 analyzer. The sample pore size distribution was characterized using the Barrett–Joyner–Halenda model. The chemical composition and valence state of the metal elements in the sample were investigated using XPS (ESCALAB‐MKII) (*hυ* = 1486.6 eV). Morphological and elemental mapping of the samples were analyzed by field emission SEM (S4800.HITACHI). TEM bright field images of the samples were recorded using a Tecnai G^2^ F30 S‐Twin microscope operated at an acceleration voltage of 300 kV. The samples were deposited on SiO_2_ TEM grids (TEM windows, USA). TEM imaging at room temperature was performed using a double tilt cooling holder (Gatan, USA). Grazing‐incidence wide‐angle X‐ray scattering (GIWAXS) measurements were also performed at the 3C‐SAXSl beam line in the Pohang Accelerator Laboratory using a monochromatized X‐ray radiation source of 10.55 eV (*λ* = 0.117 nm) and a 2D charge‐coupled device (CCD) detector (Mar165 CCD). The NiV_2_O_6_/AgMn samples were mounted on a *z*‐axis goniometer equipped with a vacuum chamber (≈10^−3^ Torr). The samples were 0.201 m away from the CCD detector. The incident angle of each X‐ray beam was set as 0.1°, and the scattering angles were determined from the positions of the reflected X‐ray beam from the silicon substrate using precalibrated silver behenate.

### Electrochemical OER and ORR Activities of Dual‐Phase NiV_2_O_6_/AgMn

The electrocatalytic OER experiments were conducted with an electrochemical workstation (Interface1010 potentiostat/galvanostat/ZRA) with a three‐electrode setup. The sample prepared on a substrate was used as a working electrode (active area of 0.2 cm^2^). A graphite rod and Hg/HgO (NaOH, 1 m) were used as the counter and reference electrodes, respectively. The potential was measured relative to the Hg/HgO electrode followed by calibration to the RHE according to the Nernst equation: *E*_RHE_ = *E*_Hg/HgO_ + 0.059 × pH + *E* ^0^. LSV and Tafel plots were iR corrected with an iR drop compensation level of 95%. iR compensations were determined from the equation *E* (iR drop correction) = *E* (uncorrected) − *I* (electrode) × *R*, where *I* is the current data of the working electrode and *R* is the module of the ohmic drop electrochemical workstation tested with ohmic drop compensation. EIS measurements were performed on a standard potentiometer equipped with a Nova impedance spectrum analyzer (AUTOLAB/PGSTAT, 128N) within a frequency range of 0.10 mHz to 100 kHz ±5 mV (KOH, 1 m).

The electrochemical ORR activity of the samples was measured by a RDE (BioLogic Science Instruments) connected to a DY 2300 potentiostat. A three‐electrode assembly in 0.1 m KOH solution used a sample‐coated glassy carbon electrode (GC; 0.196 cm^−2^), a graphite rod, and Hg/HgO as the working electrode, counter electrode, and reference electrode, respectively. The working electrode catalyst ink was prepared by dispersing 3 mg of catalyst in 0.75 mL of H_2_O, 0.25 mL of propanol, and 8 µL of 5% ethanol Nafion solution by sonication for 30 min. After that, a loading of 0.23 mg cm^2^ was achieved by drop‐casting 3 µL of the abovementioned ink onto the prepolished carbon disk electrode. Before each RDE run, the KOH electrolyte solution was saturated with oxygen for 1 h. The discharge polarization curve and peak power density of each battery were measured using a Biologic potentiostat (Biologic, VMP‐3) device. The discharge performance of each cell was evaluated using the Wonatech Cycler System (Wonatech, WBCS3000). For Zn‐air battery module, pouch, and coin cell tests, batteries were tested at room temperature for a 10 mA cm^−2^ current density.

## Conflict of Interest

The authors declare no conflict of interest.

## Supporting information

Supporting Information

## Data Availability

The data that support the findings of this study are available from the corresponding author upon reasonable request.
