# Supplementary material for: Hydrothermally Grown Dual‐Phase Heterogeneous Electrocatalysts for Highly Efficient Rechargeable Metal‐Air Batteries with Long‐Term Stability
Source: Adv Sci (Weinh). 2022 Sep 14;9(32):2203663. doi: 10.1002/advs.202203663 (PMC9661842; doi:10.1002/advs.202203663)
Supplement: Supplementary file 1 — Supporting Information [file ADVS-9-2203663-s001.pdf]

ADVANCED SCIENCE
Open Access

Supporting Information

for *Adv. Sci.*, DOI 10.1002/advs.202203663

Hydrothermally Grown Dual-Phase Heterogeneous Electrocatalysts for Highly Efficient Rechargeable Metal-Air Batteries with Long-Term Stability

*Chandran Balamurugan, Changhoon Lee, Kyusang Cho, Jehan Kim, Byoungwook Park, Yusin Pak, Jaemin Kong and Sooncheol Kwon**

Supporting Information

Hydrothermally grown dual-phase heterogeneous electrocatalysts for highly efficient rechargeable metal-air batteries with long-term stability

*Chandran Balamurugan, Changhoon Lee, Kyusang Cho, Jehan Kim, Byoungwook Park, Yusin Pak, Jaemin Kong, and Sooncheol Kwon**

Dr. C. Balamurugan, Prof. S. Kwon
Department of Energy and Materials Engineering, Dongguk University-Seoul, Seoul, 04620 Republic of Korea
Email: kwansc12@dongguk.edu

Dr. C. Balamurugan
Heeger Center Advanced Materials (HCAM), Gwangju Institute of Science and Technology (GIST), Gwangju 500-712, Republic of Korea.

Dr. C. Lee
Max Planck POSTECH Center for Complex Phase of Materials, Pohang University of Science and Technology, Pohang 37673, Korea

K. Cho
Research Institute for Solar and Sustainable Energies (RISE), Gwangju Institute of Science and Technology (GIST), Gwangju 500-712, Republic of Korea

Dr. J. Kim
Pohang Accelerator Laboratory, Pohang University of Science and Technology, Pohang 37673, Republic of Korea.

Dr. B. Park
Division of Advanced Materials, Korea Research Institute of Chemical Technology, Daejeon 305-600, Republic of Korea.

Dr. Y. Pak
Sensor System Research Center (SSRC), Korea Institute of Science and Technology (KIST), Seoul 02792, Republic of Korea.

Prof. J. Kong
Department of Physics, Gyeongsang National University, Jinju 52828, Republic of Korea

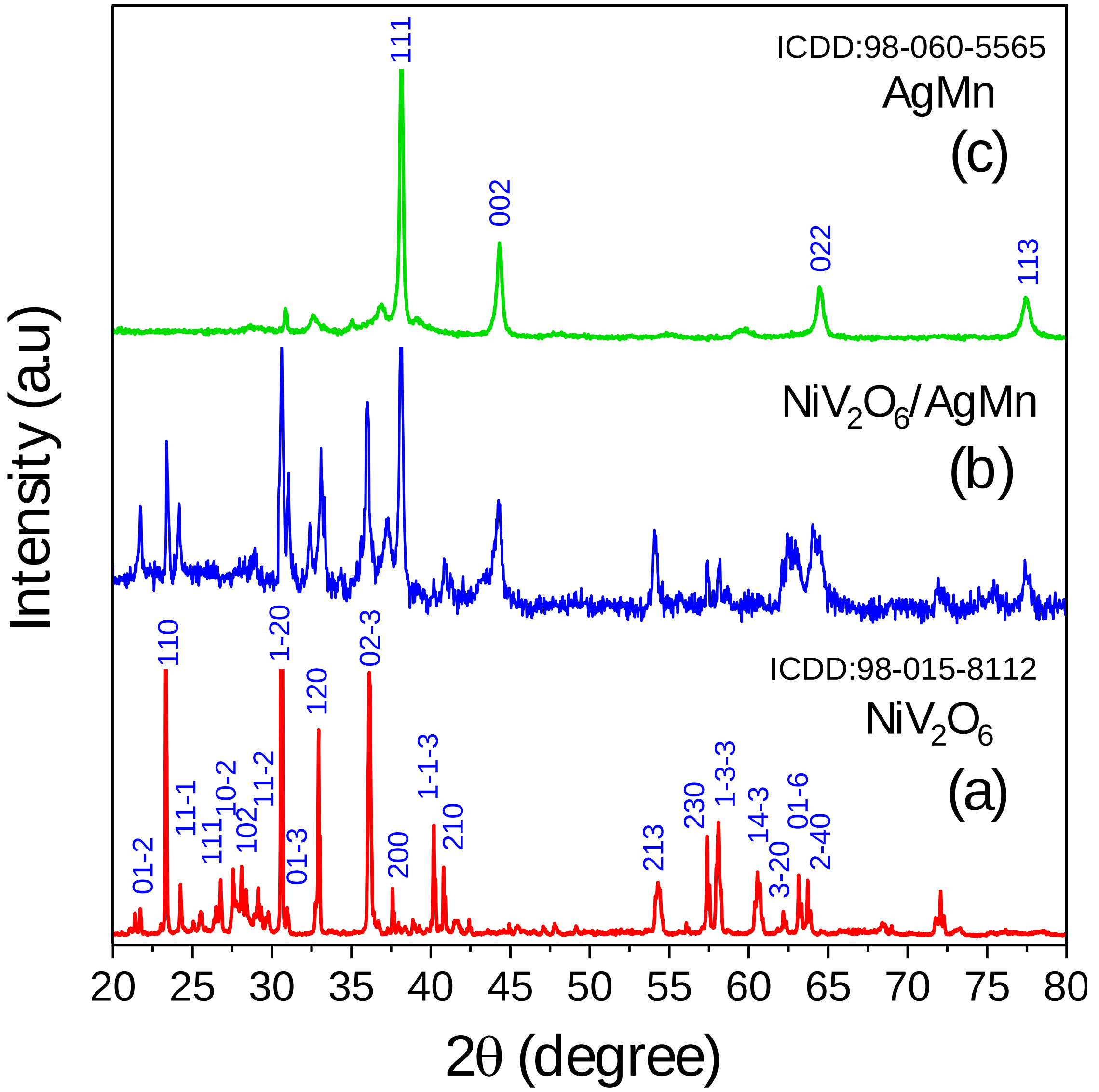

Figure S1. (a, b and c) X-ray diffraction patterns of the NiV_2O_6, NiV_2O_6/AgMn and AgMn electrocatalysts.

The crystalline properties of NiV_2O_6 prepared by the hydrothermal method were analyzed by X-ray diffraction (XRD). All peaks are consistent with the standard pattern of the triclinic crystal structure of NiV_2O_6 (ICDD-PDF file 98-015-8112), confirming the high-quality formation of the NiV_2O_6 sample (**Figure S1a**). **Figure S1c** shows the XRD pattern of the AgMn sample (ICDD-PDF file 98-060-5565). **Figure S1b** shows the XRD pattern of AgMn metal particle-decorated NiV_2O_6/AgMn, including the main characteristic diffraction peaks of NiV_2O_6 at 21.7°, 23.3°, 24.1°, 30.6°, 32.3°, 33.1°, 36.0°, 37.2°, 40.8°, 54.0°, 57.5°, 58.1°, 62.4°

and 71.9°, and the other residual peaks (38.1°, 44.2°, 64.4°, 71.9° and 81.5°) belong to the AgMn heterometal nanoparticles. In addition, the inclusion of metal nanoparticles in the NiV_2O_6 sample slightly shifted the 2θ peak compared to the NiV_2O_6 and AgMn samples, indicating lattice expansion due to metal particle substitution.

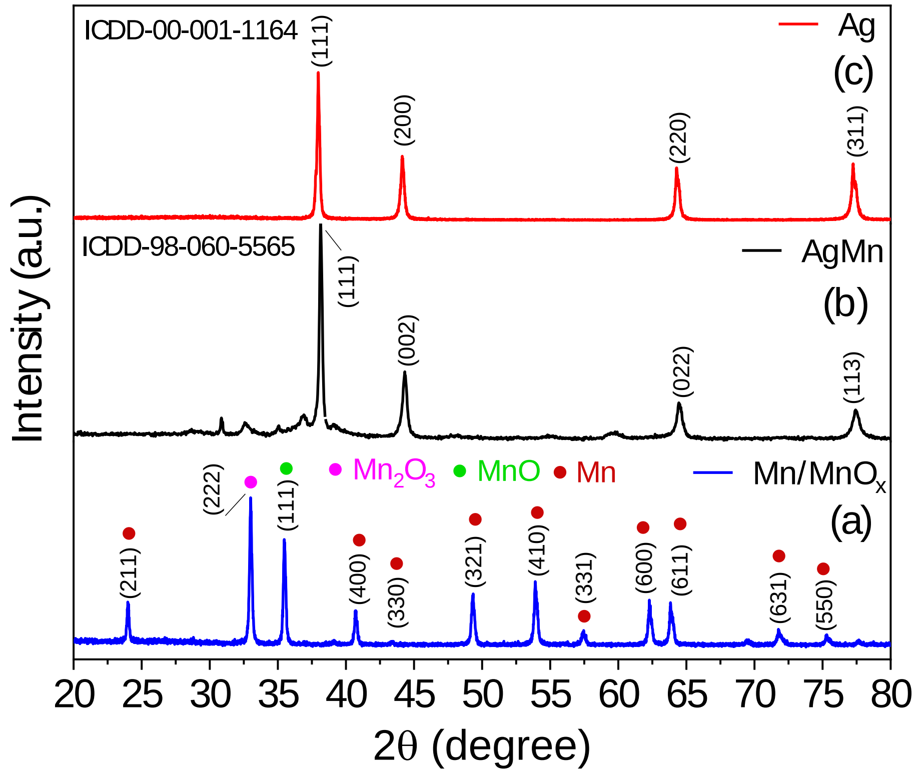

Figure S2. X-ray diffraction patterns of (a) Mn/MnO_x, (b) AgMn and (c) Ag metal nanoparticles.

Figure S2 shows the X-ray diffraction (XRD) patterns of the pristine silver (Ag) metal nanoparticles and Mn-O_x and AgMn heterometal nanoparticles. The diffraction patterns (**Figure S2a**) show characteristic peaks at approximately 24.1°, 40.6°, 43.3°, 48.9°, 54.0°, 57.1°, 62.5°, 64.1°, 71.8° and 75.2°, which correspond to the cubic (211), (400), (330), (321), (410), (331), (600), (611), (631) and (550) planes of Mn (ICDD file no: 00-020-0180, 00-021-0547, 00-032-0637). Except for Mn, partially oxidized forms of Mn_2O_3 (32.9°, (111), ICDD file no: 00-002-0896) and MnO (35.4° (111), ICDD file no: 00-075-0257) also appeared. The Ag metal nanoparticles (**Figure S2c**) contained the cubic phase (ICDD file no: 00-001-1164). **Figure S2b** shows the Ag and Mn-O_x crystalline phase connectivity of the cubic AgMn heterometal

nanoparticles (ICDD Card No-98-060-5565). XRD analysis of the AgMn sample suggested that only a fraction of the Mn metal was converted to oxides during the annealing process. In addition, the connection of Ag and Mn metal nanoparticles in the AgMn sample slightly shifted the 2θ peak compared to the Ag and Mn samples.

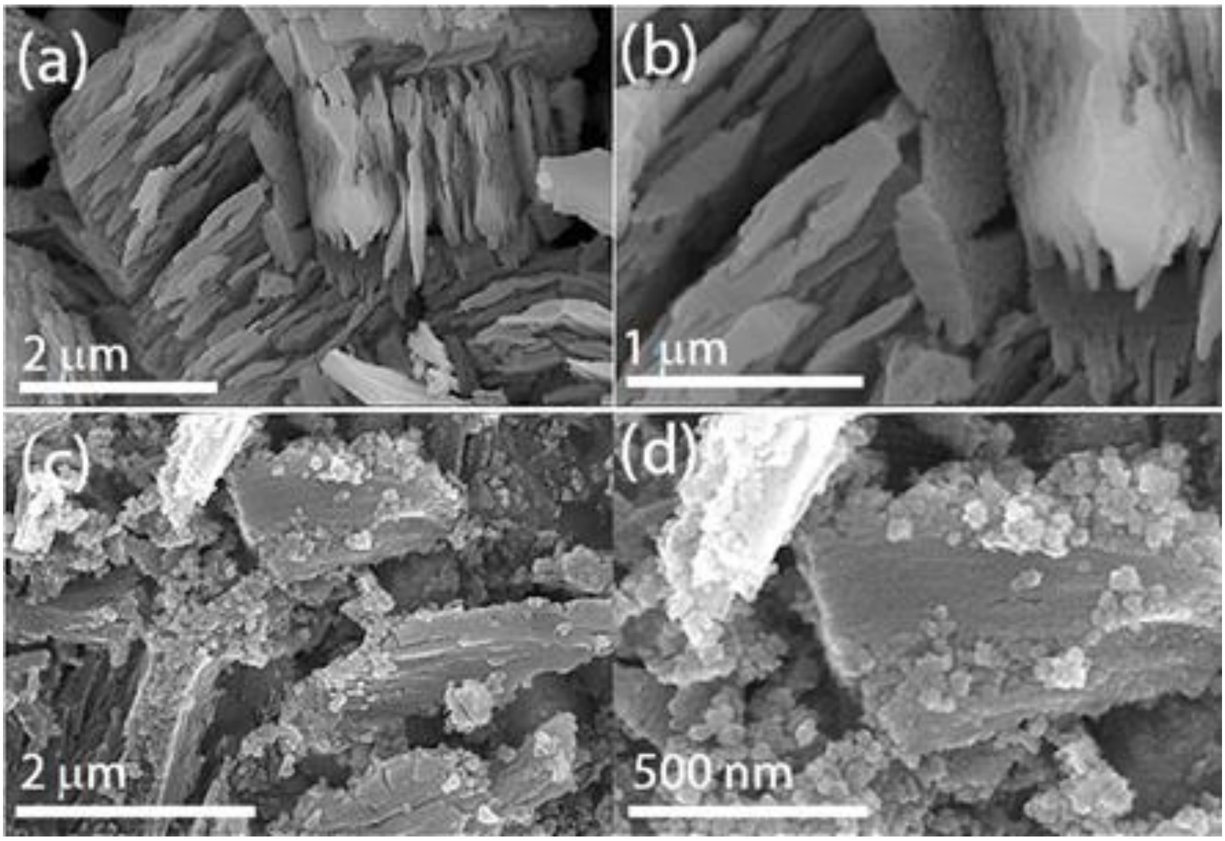

Figure S3. (a and b) Low- and high-magnification SEM images for NiV_2O_6; (c and d) Low- and high-magnification SEM images for NiV_2O_6/AgMn electrocatalysts.

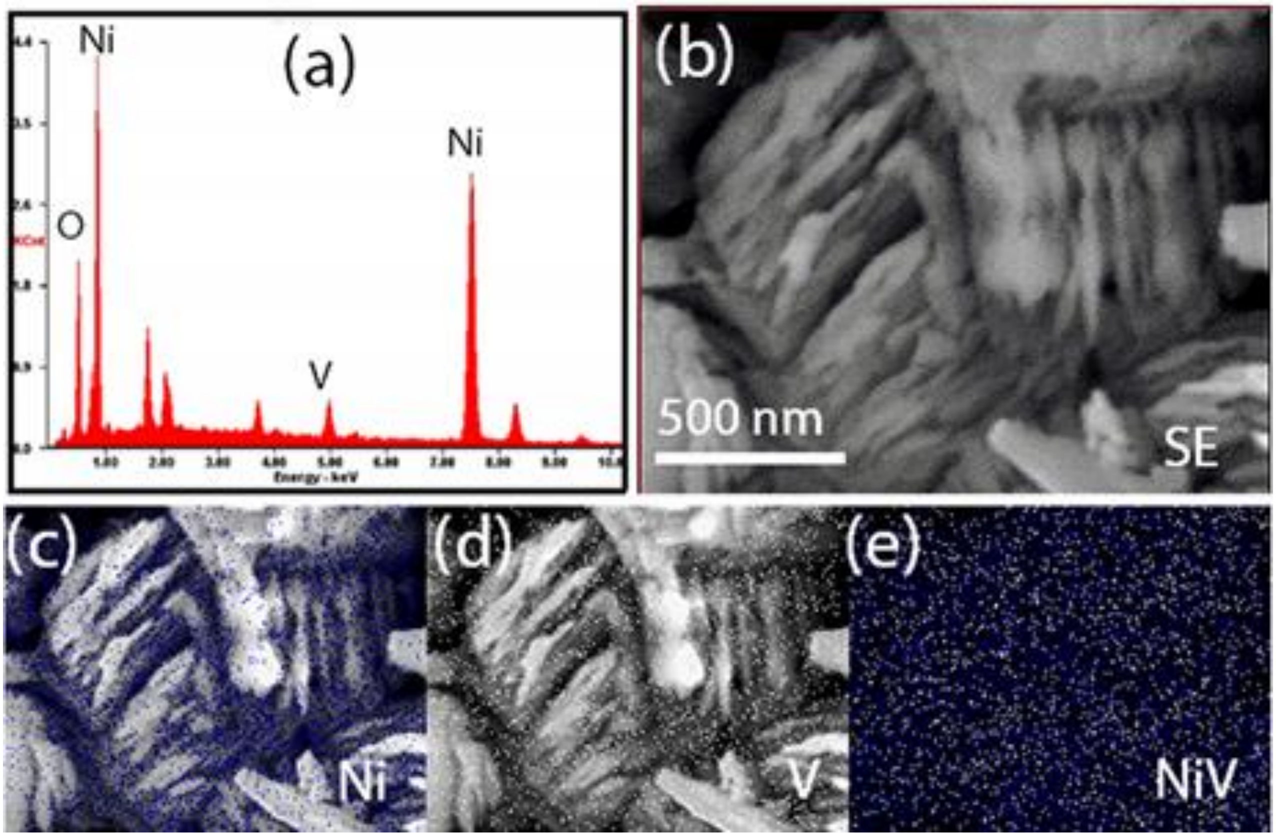

Figure S4. (a) EDS spectrum for NiV_2O_6 electrocatalysts and (b, c, d and e) the corresponding elemental mapping of Ni, V, Ag, Mn, O and mixed elements; the scale bar is 700 nm.

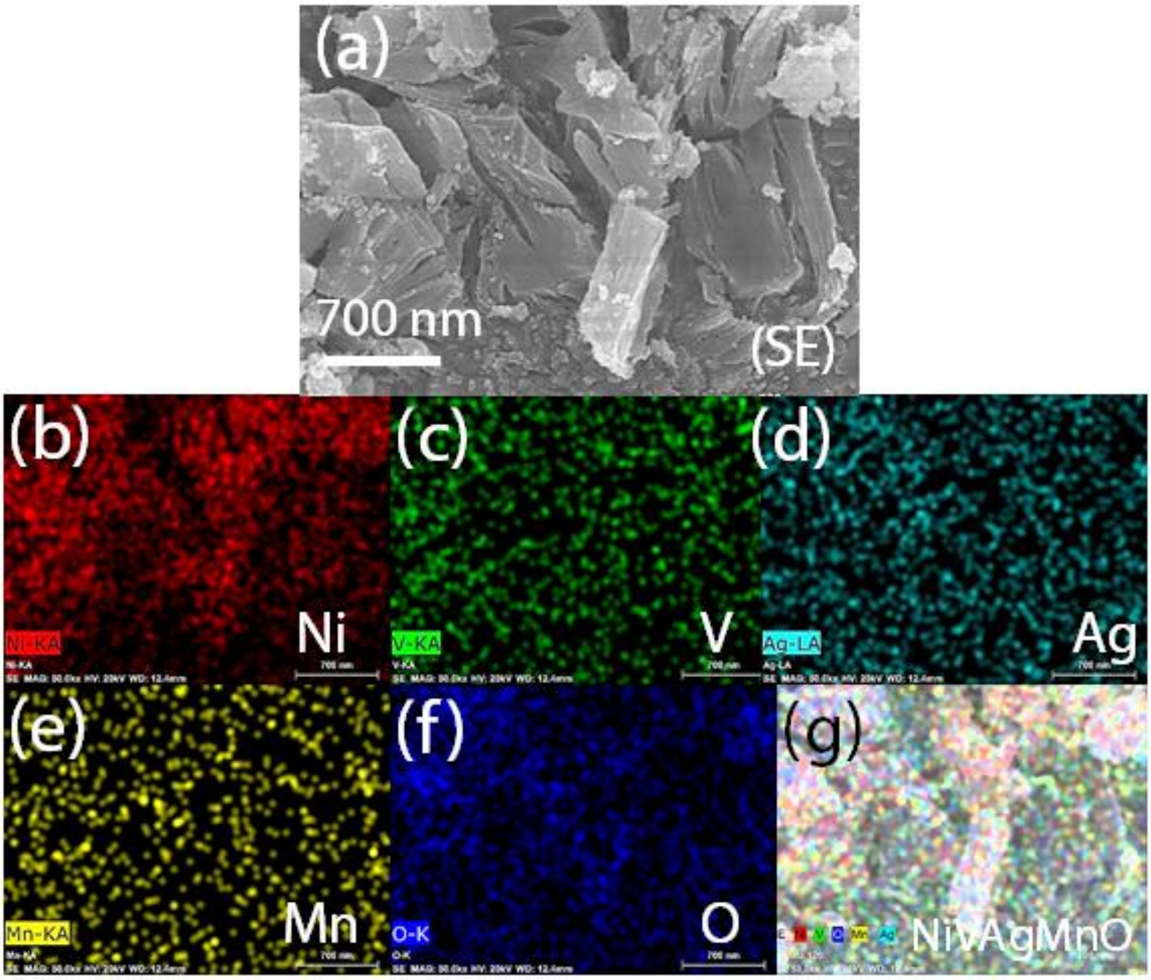

Figure S5. (a) SEM image of NiV_2O_6/AgMn electrocatalysts and (b, c, d, e, f and g) the corresponding EDS elemental mapping of Ni, V, Ag, Mn, O and mixed elements; the scale bar is 700 nm.

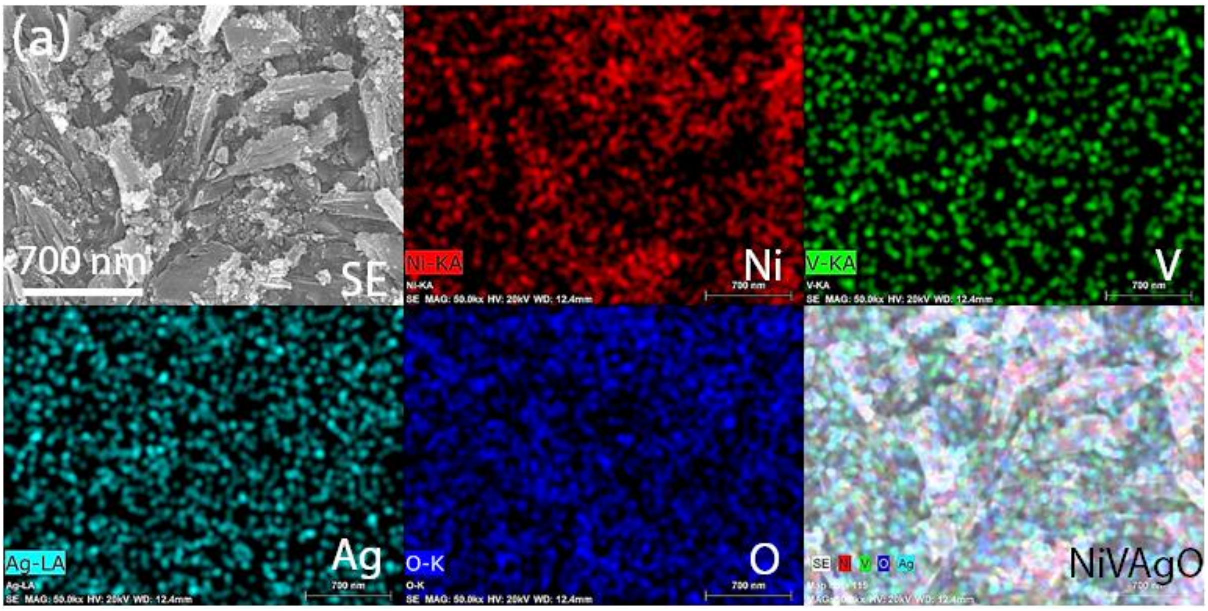

Figure S6. (a) SEM image of the NiV_2O_6/Ag electrocatalyst and the corresponding EDS elemental mapping of Ni, V, Ag, O and mixed elements; the scale bar is 700 nm.

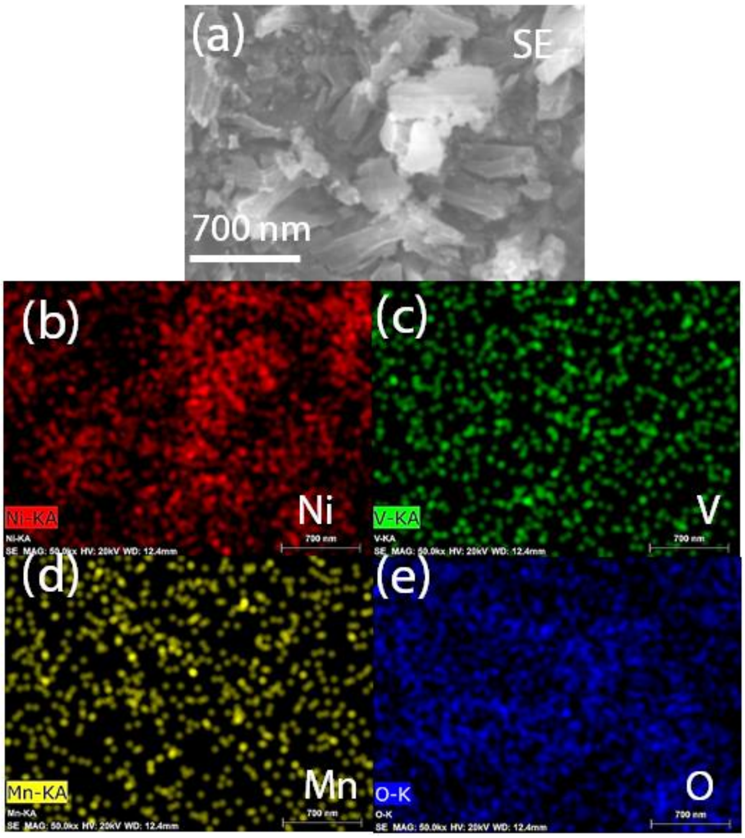

Figure S7. (a) SEM image of the NiV_2O_6/Mn electrocatalyst and (b, c, d and e) the corresponding EDS elemental mapping of Ni, V, Mn and O; the scale bar is 700 nm.

The surface morphologies of the NiV_2O_6 and NiV_2O_6/AgMn samples were analyzed by scanning electron microscopy (SEM), with the images shown in Figure S3. Low magnification images of NiV_2O_6 show that the obtained structures consist of regularly ordered 2D sheet-like structures with open porous properties. Each 2D sheet is arranged by preferentially aligned nucleation and is uniformly connected with its neighbors and packed onto the entire substrate. The high magnification image clearly shows the surface of a 2D sheet composed of spherical particles. Through EDX spectral data and element mapping analysis, it was confirmed that the two-dimensional sheet-like structure was composed only of Ni, V, and O, and no other impurities were found on the surface (Figure S4). The SEM images after AgMn metal particle decoration clearly show that the nanosheet structure remains unchanged even after the second heat treatment and that the surface of the 2D sheet is decorated with spherical-like particles. EDX elemental mapping images of NiV_2O_6/AgMn confirm the coexistence of uniformly decorated AgMn metal nanoparticles on the entire NiV_2O_6 2D sheet-like surface (Figure S5), presumably due to metal grain decoration under hydrothermal conditions. For the NiV_2O_6/Ag and NiV_2O_6/Mn samples, the SEM images and their elemental mapping clearly reveal Ag and Mn metal nanoparticle decoration on the NiV_2O_6 surface (Figure S6, S7).

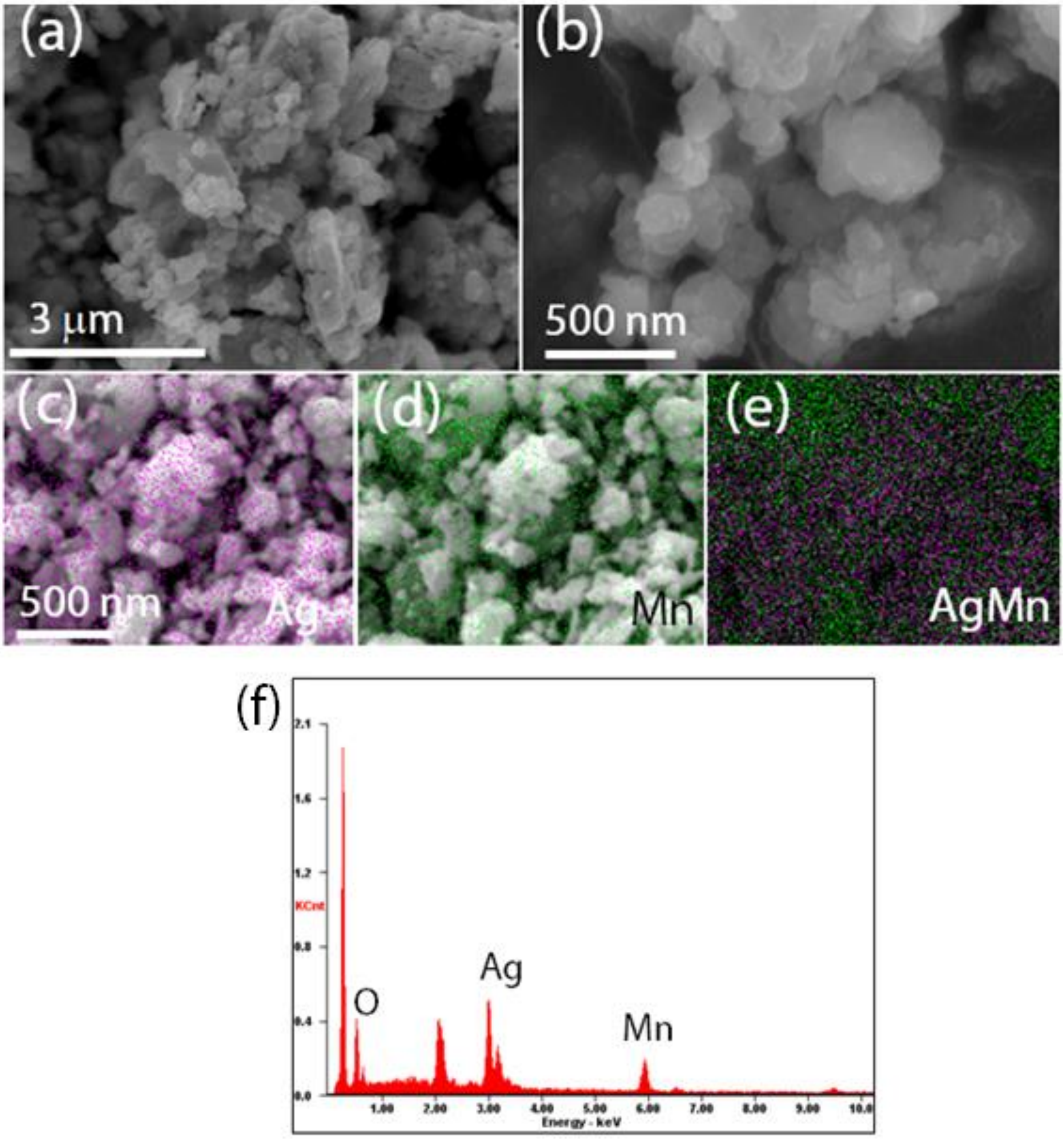

Figure S8. (a and b) Low- and high-magnification SEM images of AgMn nanoparticles and the (c, d and e) corresponding EDS elemental mapping of Ag, Mn, O and mixed elements; the scale bar is 500 nm. (f) EDS spectrum for AgMn nanoparticles showing the presence of Ag, Mn and O.

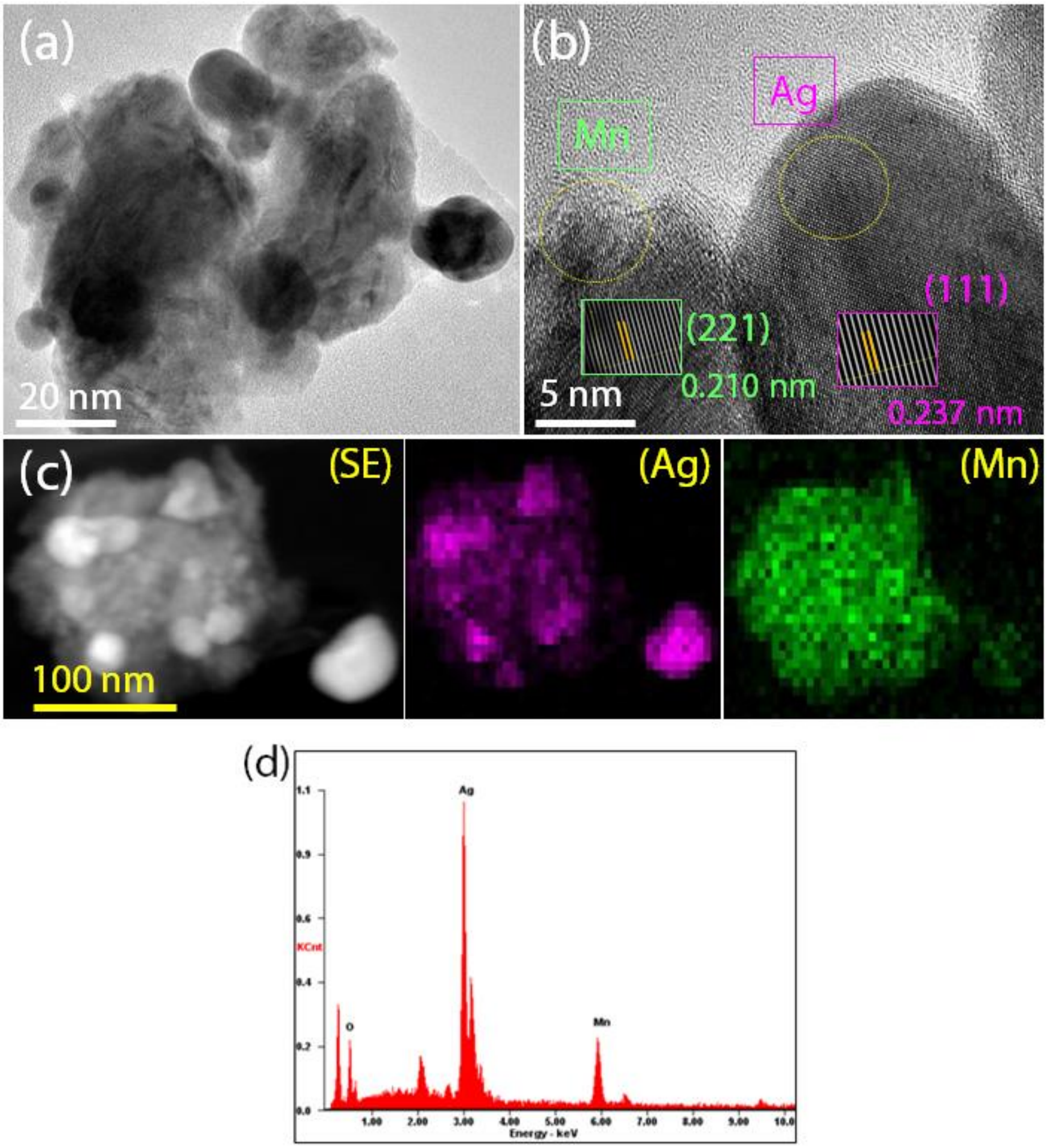

Figure S9. (a) TEM image of encapsulated AgMn nanoparticles, (b) HRTEM images of the AgMn nanoparticles and corresponding magnified lattice fringes (inset) of Ag and Mn, (c) STEM image of AgMn and the related elemental mapping of Ag and Mn, and (d) corresponding EDS spectrum for AgMn nanoparticles.

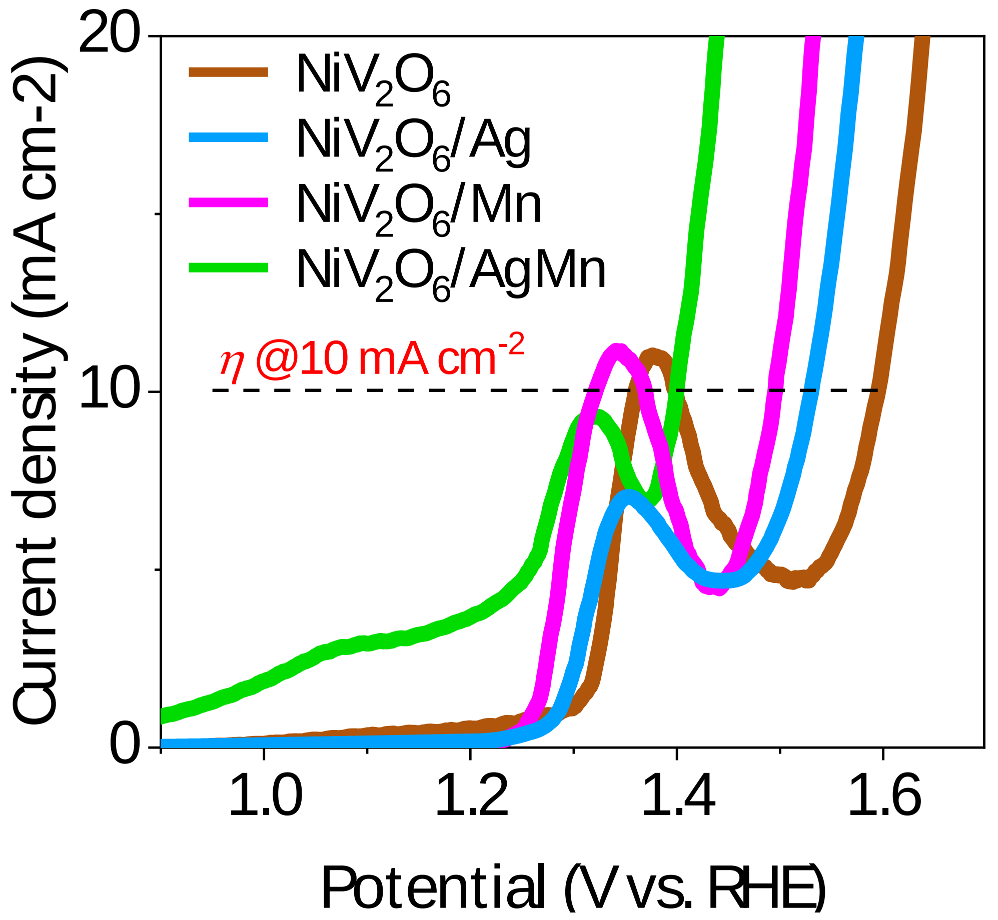

Figure S10. The enlarged portion of the LSV shows Ni^{2+} to Ni^{3+} or $^{4+}$ peaks in the NiV_2O_6, NiV_2O_6/Ag, NiV_2O_6/Mn and NiV_2O_6/AgMn catalysts.

The oxygen evolution reaction of Ni-based catalysts in KOH electrolytes with $Ni^{3+ \text{ or } 4+}$ oxidation intermediates is considered the active site of the OER.[1-5] Therefore, the $Ni^{+3 \text{ or } +4}$ oxidation peaks were investigated to explain the enhanced oxygen evolution capacity of the catalysts. The observed anodic peak at 1.38 V vs. RHE for NiV_2O_6 is probably related to the oxidation of Ni^{2+} to $Ni^{3+ \text{ or } 4+}$. The anodic oxidation peak of Ag metal particles with the NiV_2O_6 catalyst shifted to 1.35 V toward the cathode. A similar behavior was observed for the NiV_2O_6/Mn catalyst, and the oxidation peak shifted negatively to 1.34 V vs. RHE. However, the decoration of AgMn bimetallic particles has a significant effect on the NiV_2O_6 surface, which more negatively shifts Ni^{2+} to the $Ni^{3+ \text{ or } 4+}$ oxidation peak at 1.31 V vs. RHE. This may be because the incorporated metal particles can affect Ni oxidation potentials by changing the local electronic environment around Ni-O-V. Moreover, compared to the NiV_2O_6/Ag and

NiV_2O_6/Mn catalysts, NiV_2O_6/AgMn showed a greater cathodic shift because the dual-metal content of the catalyst could increase the degree of distortion in the local Ni-O-V environment, thereby increasing the catalytic activity.[6]

Furthermore, due to the strong electron interaction between the AgMn metal particles and the NiV_2O_6 catalyst, the oxidation peak current of the NiV_2O_6/AgMn catalyst is suppressed because the catalytic OER onset potential appears much earlier than this oxidation peak.[7] Moreover, NiV_2O_6/Ag and NiV_2O_6/Mn catalysts decorated with single metals showed lower OER activity than NiV_2O_6/AgMn catalysts due to the small contact areas and weak interaction between the single metals. The superior performance of NiV_2O_6/AgMn is based on two indispensable metal particles rather than any one. Therefore, the NiV_2O_6/AgMn catalyst is characterized by a more negative OER active oxidation peak due to the strong interaction of multiple metal particles with various heterointerfaces, such as AgMn and/or MnAg, which plays a pivotal role in reducing the overpotential of the OER.

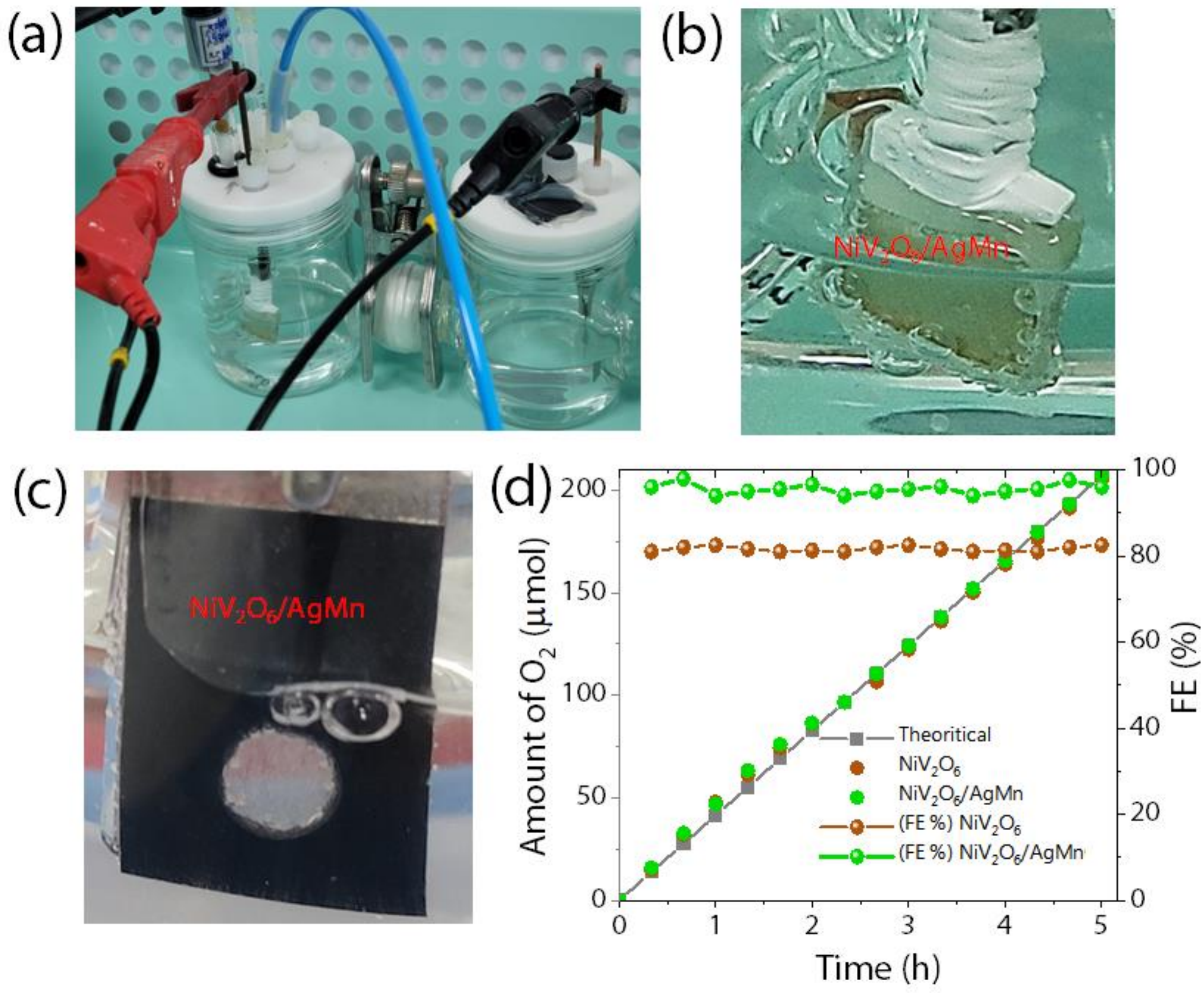

Figure S11. (a) Evolved O_2 gas measurements were estimated in a closed cell using GC (mode of field operation) to analyze the headspace. (b) The evolved O_2 gas measurement electrode masked with inert epoxy was set at approximately 1.0 cm^2. (c) LSV before measurement of electrodes masked with inert epoxy, and (d) time dependence of the Faraday efficiency of the oxygen evolution of NiV_2O_6 and NiV_2O_6/AgMn catalysts.

The evolved O_2 gas measurements were estimated in a closed cell using GC (in situ operating mode, YL Instrument 6500 GC System) to analyze the headspace. Argon (99.9998%, Ar, 20 mL/min) was purged through the cell during analysis to remove O_2 from the working electrode. Prior to analysis, the electrode masked with inert epoxy was set to approximately 1.0 cm^2 to ensure that only the area of the electrode was in direct contact with the electrolyte. The amount of O_2 released was determined by bringing the gas online from the cell headspace to the gas sampling loop of the GC every 20 minutes using a metal line.

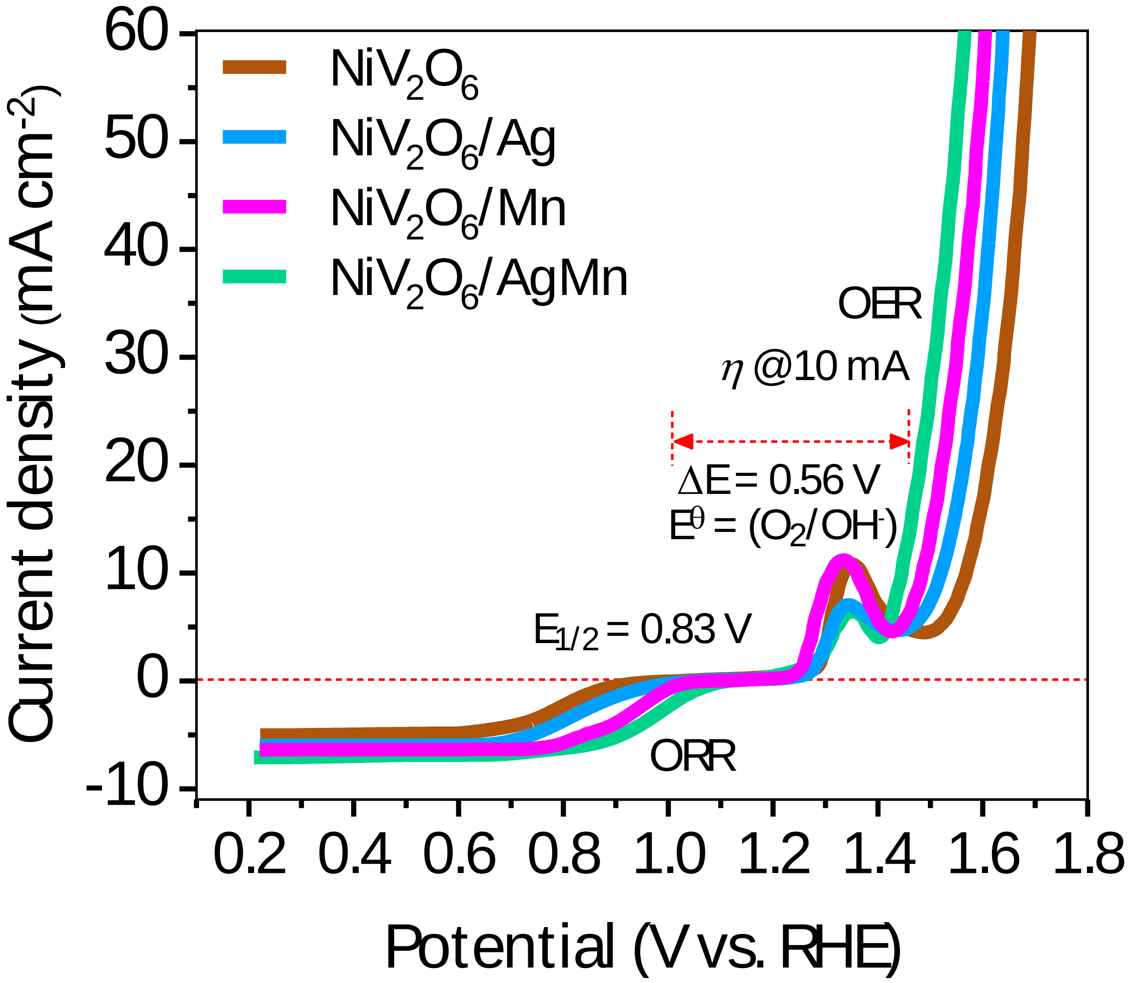

Figure S12. The LSV curves of the overall bifunctional ORR and OER activities of various catalysts in an aqueous KOH solution with a rotating speed of 1600 rpm. Inset value ΔE ($E_{j=10}$-$E_{1/2}$) 0.56 V for NiV_2O_6/AgMn.

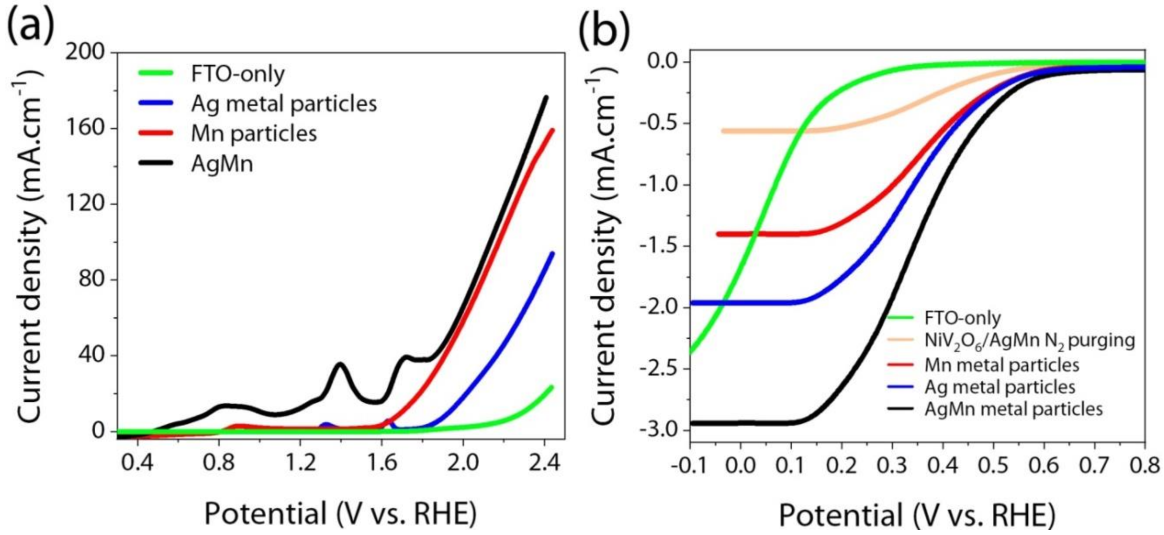

Figure S13. (a and b) OER and ORR profile curves of the FTO substrate as well as Ag, Mn, and AgMn particles and the ORR profile curve of N_2 purge conditions for the NiV_2O_6/AgMn catalyst.

For comparison, the OER and ORR electrochemical activities of Ag, Mn-O_x, and AgMn hetero-metal nanoparticles were also measured, and the results were significantly inferior to those of NiV_2O_6/AgMn.

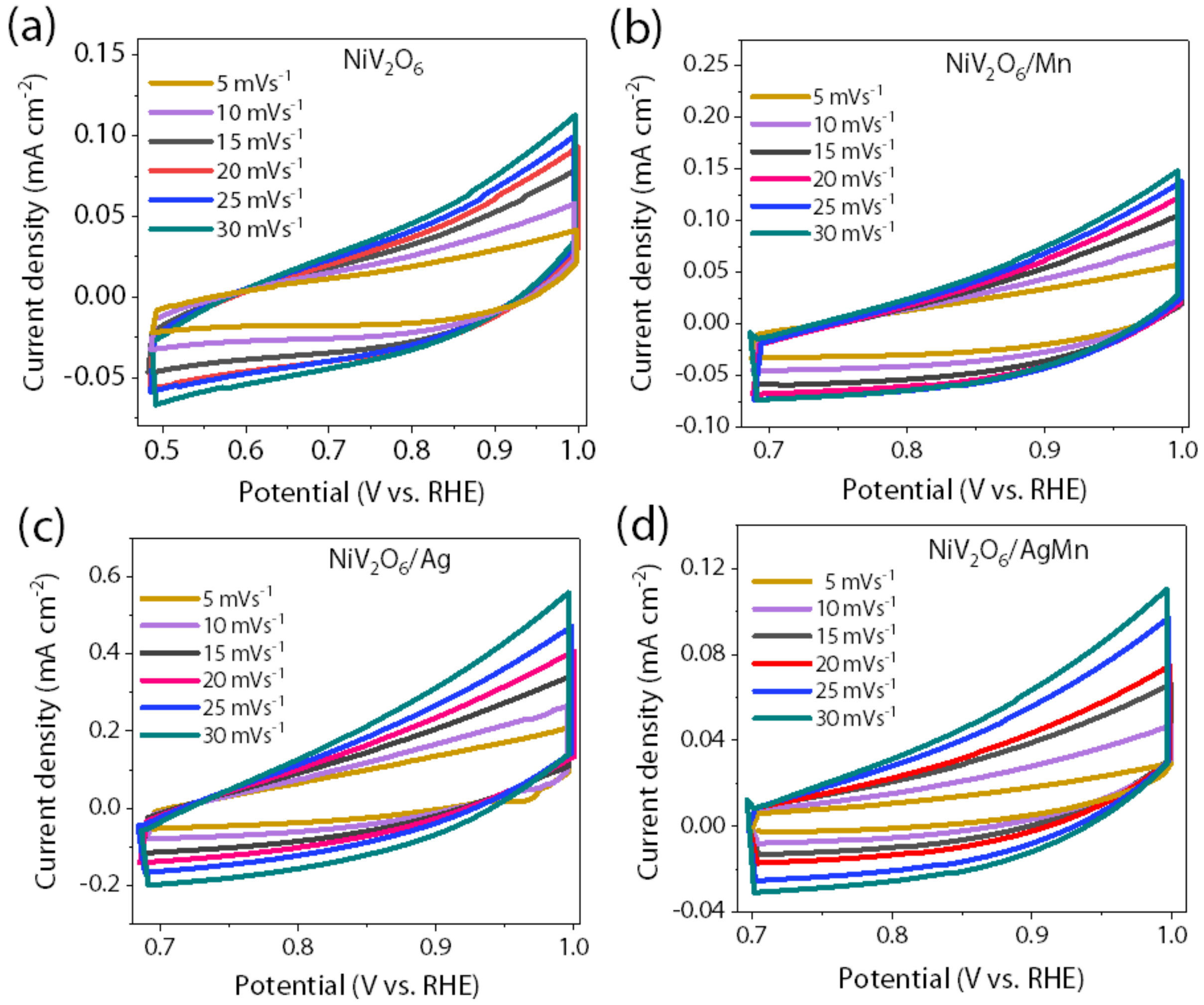

Figure S14. Cyclic voltammograms measured in the non-Faradic potential region at scan rates of 5, 10, 15, 20, 25 and 30 mVs^{-1} for (a) NiV_2O_6, (b) NiV_2O_6/Mn, (c) NiV_2O_6/Ag, and (d) NiV_2O_6/AgMn catalysts in 1 M KOH.

Electrochemically Active Surface Area (ECSA)/RF calculation for the OER:

To explore the active surface areas of catalysts, the electrochemically active surface area (ECSA) of each electrode was determined. The ECSA was evaluated using the electrochemical double-layer capacitance (C_{dI}) determined by cyclic voltammetry (CV) with varying scan rates (mV/s) in the non-Faradic potential region. Cdl measurements of the working electrode were performed at various sweep rates (5, 10, 15, 20, 25 and 30 mV s^{-1}) in N_2-saturated 1 M KOH using a CV non-Faraday zone.

The C_{di} is given by the formula:

Double layer capacitance (C_{di}) = capacitance current (*i*)/scan rate (υ)

The slope of the regression line in the plot of capacitive current (*i*) versus scan rate (υ) was used to determine the double layer capacitance (C_{di}), as shown in Figure 3h.

The ECSA was then calculated from C_{di} using the formula:

ECSA = C_{di} (mF)/Cs (µFcm^{-2})

where Cs is the specific capacitance for heterogeneous catalysts, and Cs has been found to be generally in the range of 40 µFcm^{-2} in alkaline medium according to references. Therefore, we estimated the ECSA for each catalyst using the calculated Cs of 40 µFcm^{-2}. The obtained ECSA values of NiV_2O_6, NiV_2O_6/Ag, NiV_2O_6/Mn and NiV_2O_6/AgMn were 54.7, 82.8, 102.8 and 168.0 cm^2, respectively.

The roughness factor (RF) was calculated based on the ECSA of the obtained catalyst (RF = ECSA/geometric area), and the values are summarized in Table S2.

Calculation of the turnover frequency (TOF) for the OER:

Assuming all metal atoms in the sample are active and have access to the electrolyte, the TOF values can be obtained according to the following equation:

$TOF = jNa/4FN$

where j (mA cm^{-2}) is the current density at a specific potential, NA is Avogadro's number, F is Faraday's constant (9485 C mol^{-1}), and N is the number of moles of catalytic active

material.

At an overpotential of 360 mV, the TOF of NiV_2O_6/AgMn was calculated to be 0.0225 s^{-1}, which is much higher than those of NiV_2O_6/Mn (0.0131 s^{-1}), NiV_2O_6/Ag (0.0065 s^{-1}) and NiV_2O_6 (0.0036 s^{-1}), indicating the better intrinsic activity of NiV_2O_6/AgMn.

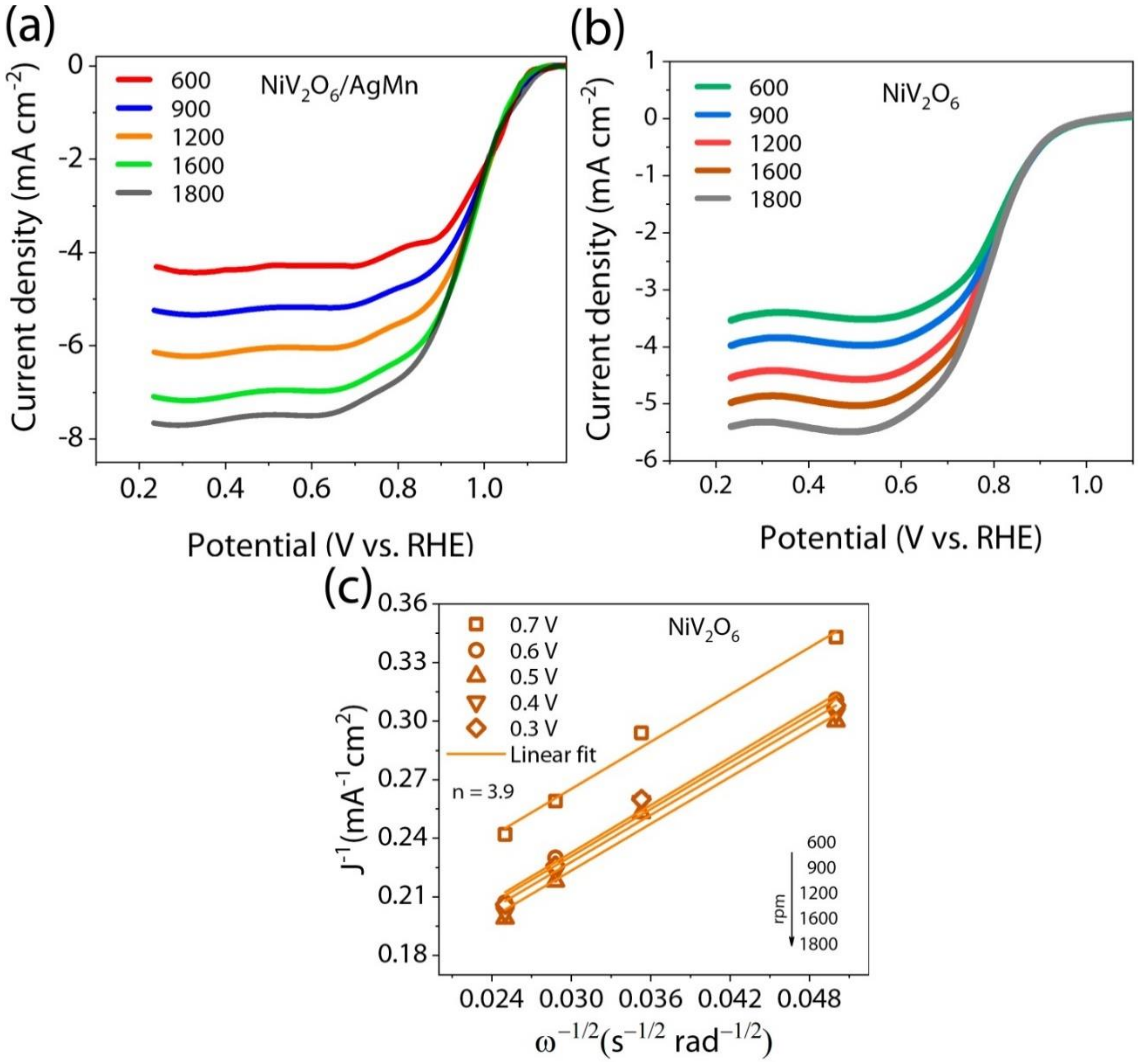

Figure S15. (a and b) ORR polarization curves of NiV_2O_6 and NiV_2O_6/AgMn catalysts at different rotation speeds from 600 to 1800 rpm at a scan rate of 5 mV s^{-1}. c) *K-L* plots obtained from polarization curves at different potentials and corresponding electron transfer numbers (*n*) of the NiV_2O_6 catalyst in oxygen-saturated 0.1 M KOH aqueous solution.

Calculation of the number of electrons transferred per oxygen molecule in the ORR:

To explore the electron transfer number (*n*) per oxygen molecule in the ORR process, LSV polarization curves with different rotation speeds (400-1800 rpm) were measured, as shown in the figure, which indicates that the current density increases with increasing oxygen diffusion. The correlation between the disk current and the kinetic current density is obtained from the *K-L* equation:

$1/j = 1/j_k + 1/j_d$

The limiting diffusion current is obtained from the following equation:

$J_d = 0.62nFAC_oD_o^{2/3}v^{-1/6}\omega^{1/2} = B\omega^{1/2}$

where *jk, jd*, D_o, *v, F*, C_o and n are the kinetic current, diffusion current, diffusion coefficient of oxygen in 0.1 M KOH (1.9×10^{-5} cm^2/s), the viscosity of 0.1 M KOH (1.0×10^{-2} cm^2/s), Faraday's constant (*96500 C*), the KOH bulk concentration (1.2×10^{-6} mol/cm^3) and number of electrons transferred in the process.

B, the slope of the *K-L* plot, can be obtained from the following.

The slope of the Koutecky-Levich (*K-L*) plot current density (J^{-1}) as a function of the inverse square root ($1/\omega^{1/2}$) of the rotational speed at different potentials gives *B,* from which (n), the number of electrons transferred in the ORR process can be determined.

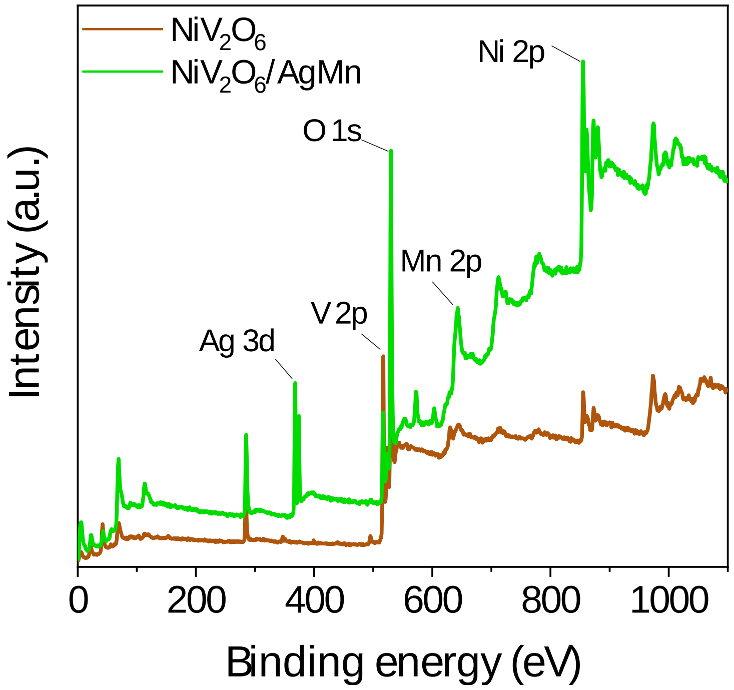

Figure S16. XPS survey spectra of NiV_2O_6 and NiV_2O_6/AgMn

The XPS survey spectra of NiV_2O_6 and NiV_2O_6/AgMn samples show the coexistence of Ni, V, O and Ni, V, Ag Mn and O elements, respectively

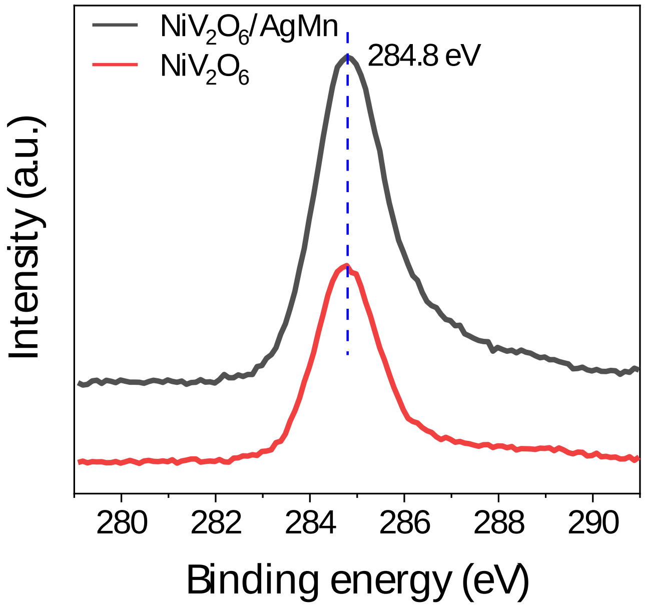

Figure S17. C 1s XPS spectra of NiV_2O_6 and NiV_2O_6/AgMn catalysts.

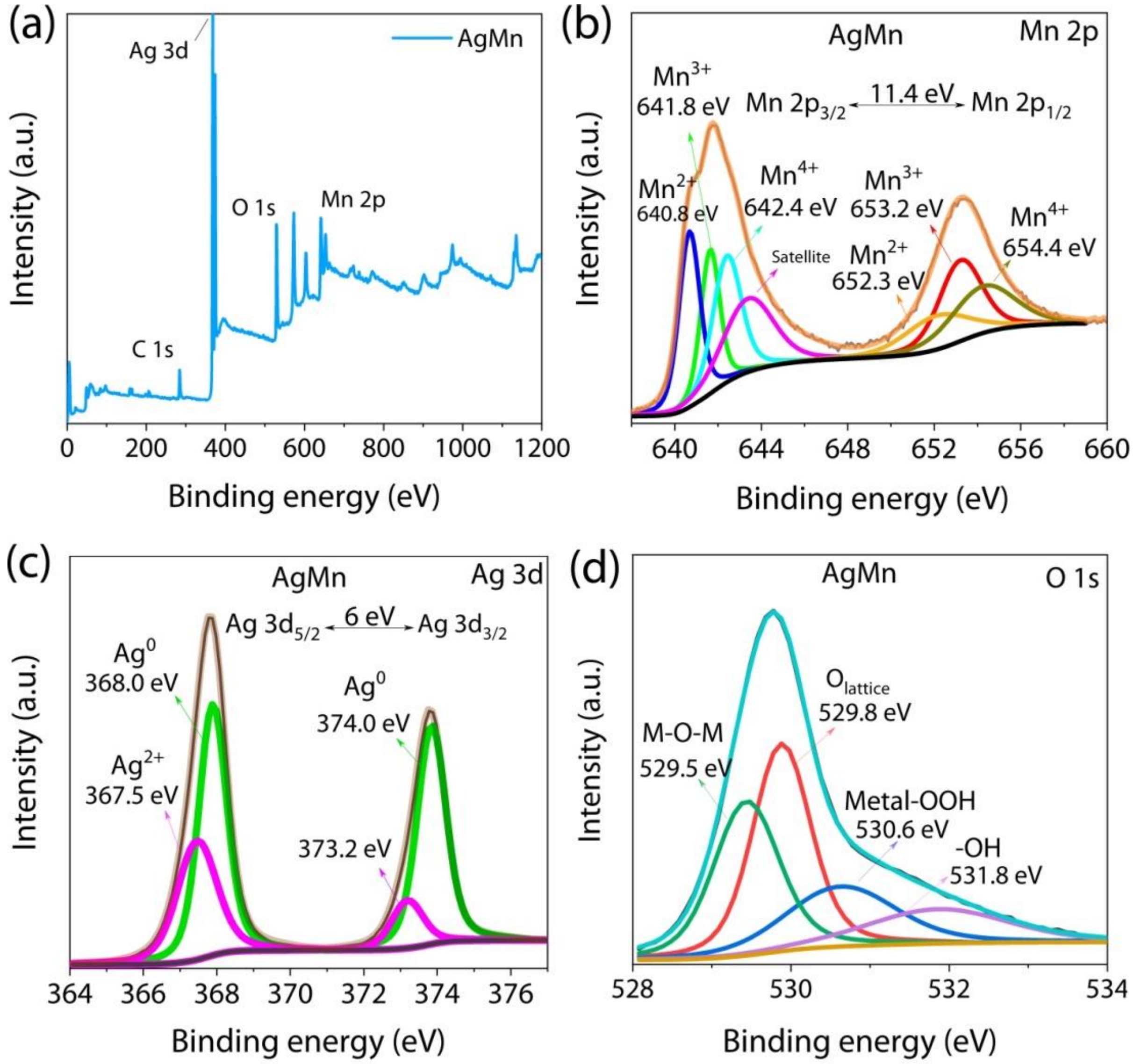

Figure S18. (a) XPS survey spectra and high resolution (b) Mn 2p (c) Ag 3d and (d) O1s spectra for AgMn metal nanoparticles.

In the high-resolution Mn 2p spectrum of the AgMn nanoparticles, peaks at 640.8/652.3 eV[8] and 641.8/653.2 eV[9, 10] are related to the spin-orbit doublets (Mn $2p_{3/2}$, Mn $2p_{1/2}$) of Mn^{2+} and Mn^{3+}, and the peak at 642.4[11] eV was assigned to Mn^{4+} along with its satellite peak at 654.4 eV (Figure S18b). After decorating the NiV_2O_6 surface with AgMn, the presence of distinct metallic Mn^0 peaks at 637.2[12] and 651.8 eV in the spin orbital Mn $2p_{3/2}$ is probably due to the reduction of some of the MnO_x to the metallic state Mn^0 (Figure 4d), which indicates a strong interaction between Ni and Mn. This intense interaction effect is further demonstrated by an additional pair of oxidation peaks of Ni^{3+} appearing at 857.6 eV and 858.0 eV in the core-

level Ni 2p spectrum of NiV_2O_6/AgMn. A detailed view of the Mn $2p_{3/2}$ region is shown in Figure 4d. Three components can be distinguished at 639.5 eV[13, 14] for Mn^{2+}, 641.5 eV[15] for Mn^{3+} and 643.9 eV[14, 15] for Mn^{4+} [11]. The binding energies of Mn^{2+} and Mn^{3+} shifted to lower values compared with the (~ 1.3 eV and 0.3 eV) pristine AgMn nanoparticles. The Mn^{4+} peak shifted to a higher binding energy compared to the (~ 1.5 eV) pristine AgMn metal particles, which indicates that Mn^{4+} was partially oxidized by the NiV_2O_6 surface due to the strong interaction. Compared with the AgMn nanoparticle sample (Figure S18c), the NiV_2O_6/AgMn sample showed a slightly negative shift in the Ag^{2+} binding energy at 0.3 eV.

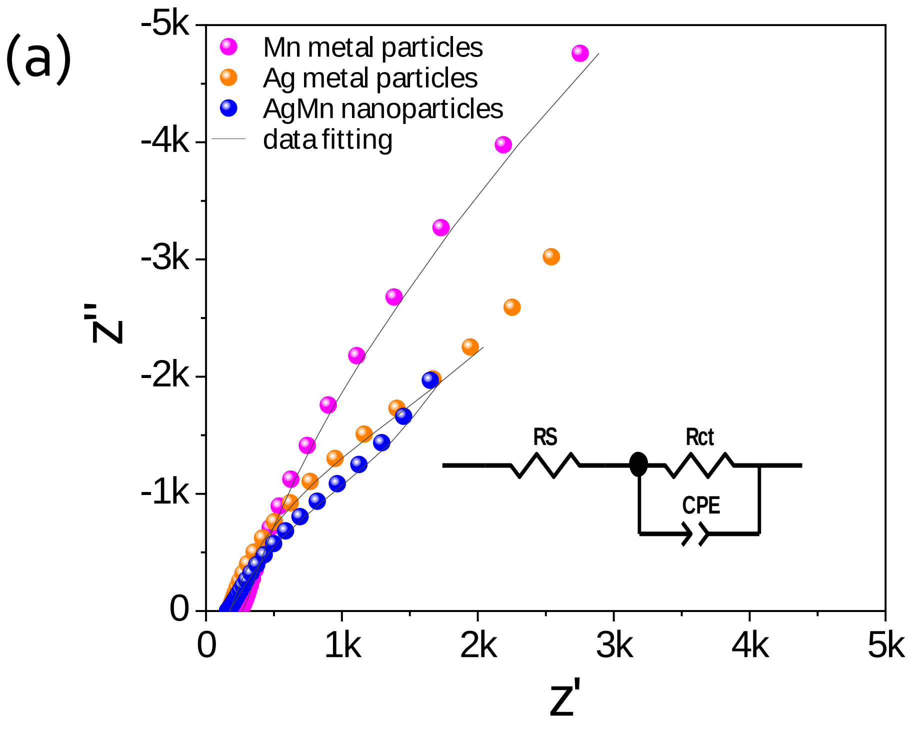

Sample	R_s (Ω)	R_{ct} (Ω)
Mn particles	46	3200
Ag particles	41	1250
AgMn particles	40	490

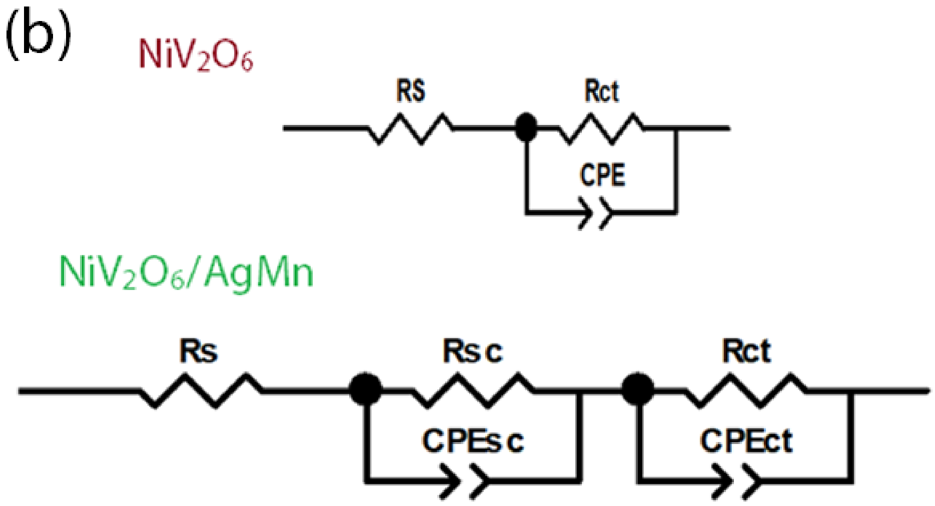

Figure S19a. Electrochemical impedance (EIS) spectra of Mn, Ag and AgMn metal particles recorded at an open circuit potential, and the inset is the equivalent circuit fitting of Nyquist plots and EIS simple Randles circuit fitted values. **(b)** Equivalent circuit fitting of Nyquist plots (simple Randles circuit). RS – solution resistance, R_{CT} – charge transfer resistance, R_{SC} – (solid–solid) charge transfer resistance and constant phase element (CPE) and corresponding fitted values.

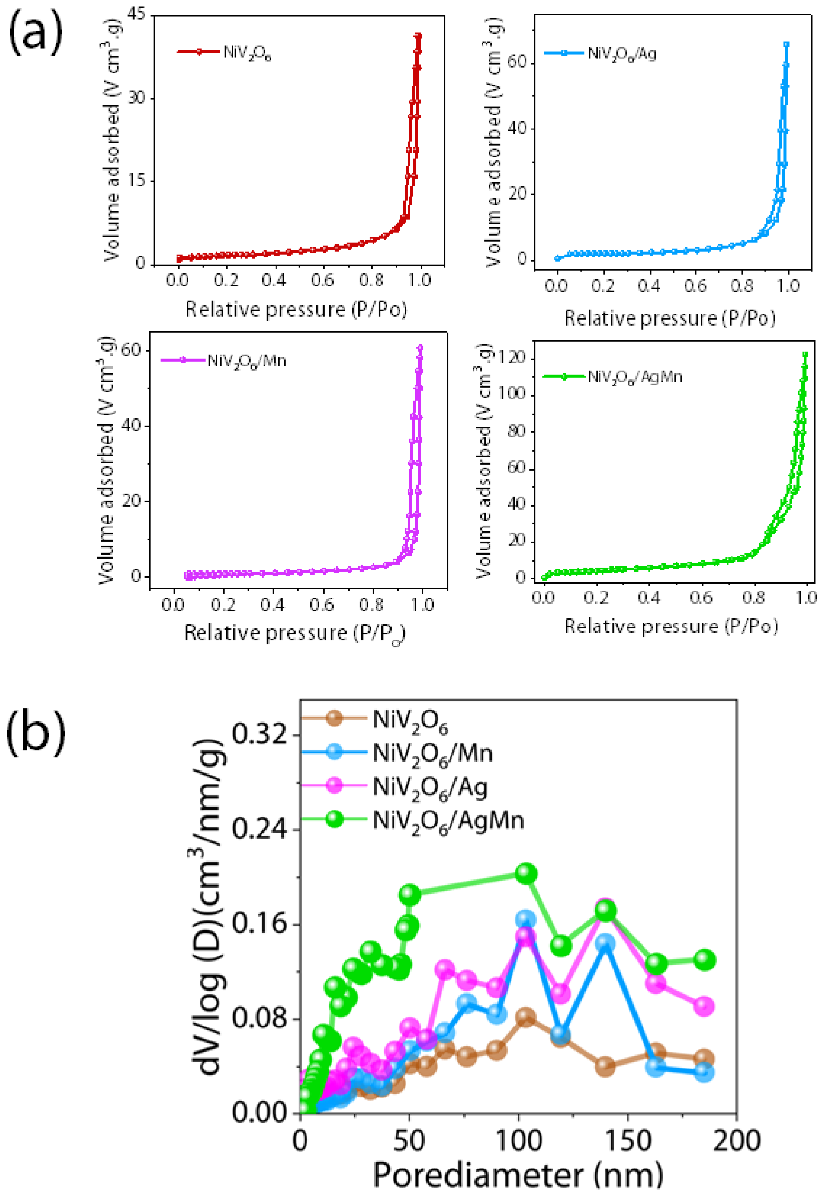

Figure S20. (a) Nitrogen adsorption/desorption isotherms of (a) NiV_2O_6, (b) NiV_2O_6/Ag, (c) NiV_2O_6/Mn and NiV_2O_6/AgMn. (b) Barrett–Joyner–Halenda pore size distribution curves of various catalysts.

The Barrett–Joyner–Halenda pore size distribution curves of the samples shows a mesoporous pore size diameter of 3-50 nm, which is consistent with the shape of the isotherm. The comparative pore volumes of the catalysts decreased from 2.90, 2.5, 2.3 and 1.9 cm^3g^{-1},

respectively. This optimized porosity of the NiV_2O_6/AgMn sample with a large surface area is favorable for its electrocatalytic dual activity.

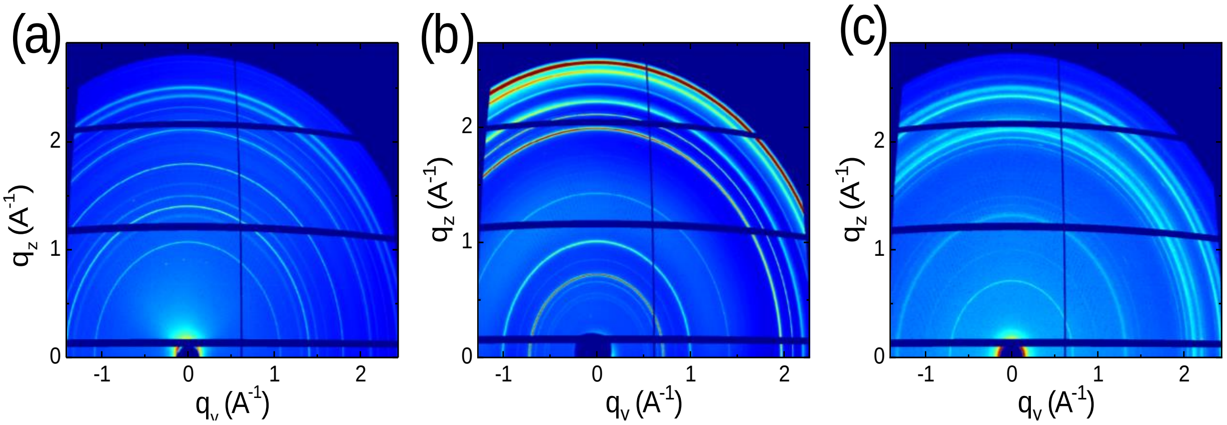

Figure S21. (a), (b) and (c) 2D GIWAX patterns of the single-phase NiV_2O_6 and AgMn and dual-phase NiV_2O_6/AgMn samples, respectively.

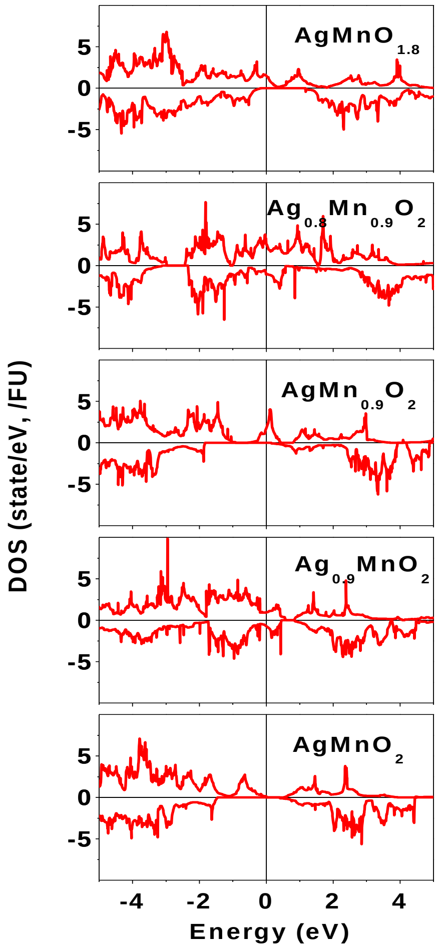

Figure S22. Comparison of the simulated density of state (DOS) of $AgMnO_2$ with different compositions.

Table S1. Comparison of the OER and ORR electrocatalytic performance of NiV_2O_6/AgMn with recently reported transition metal-based electrocatalysts.

**Catalyst**	ORR: $E_{1/2}$ **(V vs. RHE)**	**OER:** $E_{j=10}$ **(V vs. RHE)**	**ΔE=($E_{j=10}$-$E_{1/2}$) (V vs. RHE)**	Reference
CoS_2/SKJ	0.84	1.58	0.74	*ACS Nano* **2019,** *13*, 7062
Ni-MnO/RGO	0.78	1.6	0.82	*Adv. Mater.* **2018,** *30*, 1704609.
Ni_6MnO_8	0.62	1.77	1.15	*NPG Asia Mater.* **2018**, *10,* 618.
NiO/MnO_2	0.70	1.62	0.92	*ACS Appl. Mater. Interfaces,* **2017**, *9*, 42676
Ni/MnO/CNF	0.82	1.58	0.76	*Adv. Funct. Mater.* **2020**, *30,* 1910568.
$Mn_{0.5}(Fe_{0.3}Ni_{0.7})_{0.5}O_X$	0.82	1.55	0.73	*Adv. Funct. Mater.* **2020**, *30,* 1905992
$CuCo_2S_4$ NSs@N-CNFs	0.82	1.57	0.75,	*Adv. Sci.* **2019**, *6*, 1900628
$NiCo_2S_4$@NiFe LDH	0.85	1.51	0.66	*Appl. Catal. B: Environ.* **2021**, *286*, 119869
NiCo/PFC	0.79	1.63	0.84	*Nano Lett.* **2016,** *16*, 6516
Fe,Mn/N–C	0.92	1.62	0.69	*Nat Commun***, 2021,** ***12,*** 1734
N-GQDs/$NiCo_2S_4$/CC	0.86	1.57	0.71	*Small* **2019**, *15*, 1903610
FeNiCo@NC-P	0.84	1.54	0.70	*Adv. Funct. Mater.* **2019,** 30 1908167
Fe_2Ni@NC_x	0.89	1.02	0.64	*Adv. Energy Mater.* **2019**, *10* 1903003
Fe/N-G-SAC	0.9	1.61	0.71	*Adv. Mater.* **2020,** *32*, 2004900.
Ni_3FeN/Co,N-CNF	0.81	1.50	0.69	*Nano Energy* **2017,** *40* 382
NiV_2O_6/AgMn	0.83	1.39	0.56	*This work*

Table S2. Summary of intrinsic active site OER parameters of the catalyst.

Catalyst OER	η [mV] at J=10/100 mA/cm^{-2}	Tafel slope [mV·dec^{-1}]	Cdi [mF cm^{-2}]	ESCA [cm^2]	RF	TOF s^{-1} at η = 320 [mV vs. RHE]
**NiV_2O_6**	360/518	53	2.19	54.75	365	0.0025
**NiV_2O_6/Ag**	295/450	48	3.28	82.82	552	0.0065
**NiV_2O_6/Mn**	260/420	43	4.08	102.00	680	0.0123
**NiV_2O_6/AgMn**	160/345	39	6.70	168.02	1120	0.344
**RuO_2**	330/470	52	-	-	-	-

Table S3. Summary of intrinsic active site ORR parameters of the catalyst.

Catalyst ORR	Onset potential [V vs. RHE]	J_l [mA/cm^{-2}]	$E_{1/2}$ [V vs. RHE]	Tafel slope [mV.dec]	e^- *transfer number (n)*
NiV_2O_6	0.98	-4.94	0.55	79	3.9
NiV_2O_6/Mn	1.01	-5.98	0.62	64	4
NiV_2O_6/Ag	1.05	-6.29	0.79	58	4
NiV_2O_6/AgMn	1.10	-6.94	0.83	43	4
Pt/C	1.10	-4.93	0.79	61	-

Table S4. Concentrations of various oxygen species in NiV_2O_6 and NiV_2O_6/AgMn catalysts obtained from O 1s spectra.

Oxygen species	NiV_2O_6	NiV_2O_6/AgMn
Lattice oxygen species (O^{2-})	42.66%	34.55%
Highly oxidative Oxygen species (O_2^{2-}/O^-)	---	35.89%
Hydroxyl or surface adsorbed oxygen species (^-OH or O_2)	26.80%	29.56%
Surface adsorbed H_2O species	30.54%	----

Table S5. Summary of fitting parameters of Nyquist plots.

**Sample**	**Rs (Ω)**	**Rsc (Ω)**	**Rct (Ω)**
NiV_2O_6	20	-	6996
NiV_2O_6/Mn	23	121	4300
NiV_2O_6/Ag	18	35	1172
NiV_2O_6/AgMn	21	11	539

Table S6. Summary of N_2 adsorption-desorption isotherms and BJH pore size distribution parameters of NiV_2O_6 and NiV_2O_6/AgMn catalysts.

Catalysts	Pore volume cm^3g^{-1}	Average Pore diameter (nm)	BET-Surface area (m^2g^{-1})	Hysteresis loop
NiV_2O_6	2.90,	~ 48	90,	Type IV
NiV_2O_6/Ag	2.5	~ 36	120	Type IV
NiV_2O_6/Mn	2.3	~ 28	135	Type IV
NiV_2O_6/AgMn	1.9	~ 16	196	Type IV

Table S7. Summary of lattice parameters of NiV_2O_6 and NiV_2O_6/AgMn catalysts based on 2-D GIWAXS patterns.

Parameters	NiV_2O_6	AgMn	NiV_2O_6/AgMn
a(Å)	7.2	5.438	5.922
b(Å)	4.8	9.308	9.09
c(Å)	5.9	11.565	10.62
α(deg.)	98.2	113.7	122.02
β(deg.)	92.8	49.42	79.48
γ(deg.)	83.6	126.06	125.43
Volume(Å3)	200.2	359.27	387.80
Space group	1.P1	1. P1	1. P1
Crystal system	Triclinic	Triclinic	Triclinic

References:

[1] K. Xu, P. Chen, X. Li, Y. Tong, H. Ding, X. Wu, W. Chu, Z. Peng, C. Wu, Y. Xie, *J. Am. Chem. Soc.* **2015,** *137,* 4119.

[2] X. P. Wang, H. J. Wu, S. B. Xi, W. S. V. Lee, J. Zhang, Z. H. Wu, J. O. Wang, T. D. Hu, L. M. Liu, Y. Han, S. W. Chee, S. C. Ning, U. Mirsaidov, Z. B. Wang, Y. W. Zhang, A. Borgna, J. Wang, Y. H. Du, Z. G. Yu, S. J. Pennycook, J. M. Xu, *Energy Environ. Sci.* **2020,** *13,* 229.

[3] B.H.R. Suryanto, Y. Wang, R. K. Hocking, W. Adamson, C. Zhao, *Nat Commun***.** **2019,** *10,* 5599.

[4] X. Han, Y. Yu, Y. Huang, D. Liu, B. Zhang, *ACS Catal.* **2017,** *10,* 6464**.**

[5] Xiaopeng Li, Yang Wang, Jiajun Wang, Yumin Da, Jinfeng Zhang, Lanlan Li, Cheng Zhong, Yida Deng, Xiaopeng Han, Wenbin Hu, *Adv. Mater.* **2020,** 2003414.

[6] P. Li, R. Zha, H. Chen, H. Wang, P. Wei, H. Huang, Q. Liu, T. Li, X. Shi, Y. Zhang, M. Liu, X. Sun, *Small.* **2019,** *15,* 1805103.

[7] M. Qian, S. Cui, D. Jiang, L. Zhang, P. Du, *Adv. Mater.* **2017,** *29, 1704075.*

[8] X. Yan, Y. Jia, J. Chen, Z. Zhu, X. Yao, *Adv. Mater.* **2016,** *28,* 8771.

[9] D. Guo, Z. Wu, Y. An, X. Li, X. Guo, X. Chu, C. Sun, M. Lei, L. Li, L. Cao, P. Li and W. Tang, *J. Mater. Chem. C*, 2015, ***3***, 1830.

[10] Y. Zhang, P. Chen, X. Gao, B. Wang, H. Liu, H. Wu, H. Liu, S. Dou, *Adv. Funct. Mater.* **2016,** *26,* 7754.

[11] E. S. Iltona, J. E. Post, P. J. Heaney, F. T. Ling, S. N. Kerisit, *Appl. Surf. Sci.* **2016,** *366,* 475.

[12] Y. Huang, Y. Gong, J. Tang, S. Xia, *J. Hazard Mater.* **2019,** *366,* 130.

[13] D. Ji, J. Sun, L. Tian, A. Chinnappan, T.Z. W. Arachchige, D. M. Jayathilaka, R. Gosh, C. Baskar, Q. Zhang, S. Ramakrishna, *Adv. Funct. Mater.* **2020,** *30,* 1910568.

[14] V. Hiremath, M. Cho, J. G. Seo, *NewJ.Chem.* **2018,** *42,* 19608.

[15] B. Hillary, P. Sudarsanam, M. H. Amin, S. K. Bhargava, *Langmuir.* **2017,** *33,* 1743.
